# Neighborhood Properties Are Important Determinants of Temperature Sensitive Mutations

**DOI:** 10.1371/journal.pone.0028507

**Published:** 2011-12-02

**Authors:** Svetlana Lockwood, Bala Krishnamoorthy, Ping Ye

**Affiliations:** 1 School of Molecular Biosciences, Washington State University, Pullman, Washington, United States of America; 2 School of Electrical Engineering and Computer Science, Washington State University, Pullman, Washington, United States of America; 3 Department of Mathematics, Washington State University, Pullman, Washington, United States of America; University of Pittsburgh School of Medicine, United States of America

## Abstract

Temperature-sensitive (TS) mutants are powerful tools to study gene function *in vivo*. These mutants exhibit wild-type activity at permissive temperatures and reduced activity at restrictive temperatures. Although random mutagenesis can be used to generate TS mutants, the procedure is laborious and unfeasible in multicellular organisms. Further, the underlying molecular mechanisms of the TS phenotype are poorly understood. To elucidate TS mechanisms, we used a machine learning method–logistic regression–to investigate a large number of sequence and structure features. We developed and tested 133 features, describing properties of either the mutation site or the mutation site neighborhood. We defined three types of neighborhood using sequence distance, Euclidean distance, and topological distance. We discovered that neighborhood features outperformed mutation site features in predicting TS mutations. The most predictive features suggest that TS mutations tend to occur at buried and rigid residues, and are located at conserved protein domains. The environment of a buried residue often determines the overall structural stability of a protein, thus may lead to reversible activity change upon temperature switch. We developed TS prediction models based on logistic regression and the Lasso regularized procedure. Through a ten-fold cross-validation, we obtained the area under the curve of 0.91 for the model using both sequence and structure features. Testing on independent datasets suggested that the model predicted TS mutations with a 50% precision. In summary, our study elucidated the molecular basis of TS mutants and suggested the importance of neighborhood properties in determining TS mutations. We further developed models to predict TS mutations derived from single amino acid substitutions. In this way, TS mutants can be efficiently obtained through experimentally introducing the predicted mutations.

## Introduction

Temperature-sensitive (TS) mutants are fully active at permissive temperatures and less active at restrictive temperatures [1]. There are two types of TS mutants, heat-sensitive and cold-sensitive, depending on whether the permissive temperature is lower or higher than the restrictive temperature. TS mutants offer a powerful tool for *in vivo* investigation of gene function. A simple temperature shift can control gene activity and be executed in any cell type. Thus, TS mutants have been used to investigate gene function in many organisms, including viruses, bacteria, yeast, *Drosophila*, *C. elegans*, and mammalian cell cultures [2], [3], [4], [5], [6]. In fact, genetic analyses of yeast essential genes have been conducted primarily with TS mutants [7].

Despite the wide use of TS mutants in probing gene function, TS mutants are difficult to generate. The standard procedure to derive TS mutants, random mutagenesis followed by genetic screen, is time-consuming and unfeasible in multicellular organisms [8], [9]. Further, only 4–6% of all possible single amino acid substitutions in a protein are estimated to give rise to a TS mutant [10], [11], [12]. The molecular mechanisms underlying TS mutants are poorly understood. A previous crystallography study on bacteriophage T4 lysozyme suggests that TS mutations have little effect on protein structure, tend to occur at sites with low thermal factors and low solvent accessibility, and exhibit no simple pattern of amino acid substitution [13]. Mutagenesis and computational studies suggest that TS mutations can occur on buried sites or ligand-binding sites [14], [15].

Recently considerable interest has focused on applying machine learning methods to predict deleterious mutations or stabilizing mutations [16], [17], [18], [19], [20], [21], [22], [23], [24], [25], [26], [27]. However, only one study has focused on TS mutations [28]. TS mutants are very interesting because they can shift between stabilizing and destabilizing states. They are stabilizing mutations at permissive temperatures but deleterious mutations at restrictive temperatures. In this study, we applied a machine learning method, logistic regression, to investigate a large number of sequence and structure features, with the goal to elucidate the molecular basis of TS mutations. Our results indicate that neighborhood properties are important determinants of TS mutations. Assembling features also allowed us to predict single amino acid substitutions most likely to confer a TS mutation. In this way, TS mutants can be easily generated through targeted mutagenesis. This mutational engineering strategy is in principle applicable to model systems from bacteria to mammalian cell cultures, and will greatly enhance our capabilities to characterize gene functions.

## Methods

### Dataset of TS mutations

Most TS mutants used in genetic studies are heat-sensitive (the permissive temperature is lower than the restrictive temperature). Thus in our study, we focus on investigating properties of heat-sensitive mutants, which are referred to as TS mutants in the rest of the paper. More specifically, we focus on TS mutants with a single amino acid substitution (single mutants). This allows for easy mechanistic interpretation.

We assembled a set of single mutants from five proteins, on which extensive mutagenesis studies have been conducted (Table 1). These proteins include bacteriophage T4 lysozyme [10], *E. coli lac* repressor [11], *E. coli* toxin Ccdb [15], yeast TATA-binding protein (TBP) [29], and human tumor suppressor p53 [12], [30]. The crystal structures of all five proteins have been solved. For each protein, we selected a Protein Data Bank (PDB) structure [31] that had either been reported in the mutagenesis study or had the best resolution. Having access to the structure allowed us to develop and test many structure-based features. With the exception of the TBP study, which only screened for TS mutants, each of these mutagenesis studies generated both TS and neutral mutants. Neutral mutants, unlike TS, behave the same at both permissive and restrictive temperatures. Our final dataset contained 6231 single mutants, of which 747 were TS mutants and 5484 were neutral mutants. Only mutations located within the crystallized region were included. The permissive temperature of these mutants was 25°C or 30°C and the restrictive temperature was 37°C.

**Table 1:** TS and neutral mutations of five proteins.

Protein	Permissive/restrictive temperatures	PDB	TS	Neutral	Total
T4 lysozyme	25°C/37°C	2LZM_A	95	1688	1783
*lac* repressor	25°C/37°C	1EFA_A	159	2534	2693
CcdB	30°C/37°C	3VUB_A	219	1011	1230
TBP	30°C/37°C	1YTF_A	141	0	141
p53	30°C/37°C	1TUP_B	133	251	384
Total	-	-	747	5484	6231

### Sequence and structure features

We investigated 133 sequence and structure features that might be predictive for TS mutants. These features were calculated using various software programs and in-house scripts. Features fell into two categories: those describing properties of the mutation site and those describing the mutation site neighborhood (Table 2). The neighborhood is a group of residues located close to the mutated residue. We defined neighborhood based on three different distances: sequence, Euclidean, and topology. Most features have their counterparts in the three neighborhoods. Some features were derived from sequence information while others were derived from the PDB structural information. The features are described briefly here; the details are provided in Table S1.

**Table 2:** Categories of the 133 features describing single amino acid substitutions.

	Site	Neighborhood			Total
		Sequence distance	Euclidean distance	Topological distance	
Sequence-based	28	16	-	-	44
Structure-based	18	12	30	29	89
Total	46	28	30	29	133

#### Mutation site

A number of sequence conservation features were calculated using super- and subfamily alignments, including entropy, relative entropy, and the positional hidden Markov model conservation score [18]. Physicochemical properties of wild type and mutant residues were investigated, including hydrophobicity [14], volume [32], charge, Grantham values [33], and unusual residues. In addition, we classified the twenty amino acids into three groups: non-polar, polar, and charged. A number of binary features were developed based on this grouping: whether the wild type or mutant residue belongs to each of the three classes, and whether the mutation belongs to each of the nine possible substitutions. Further, we evaluated whether a mutation was located in the disordered region of a protein [34].

Solvent accessibility is the degree to which a residue is accessible to solvent molecules. We calculated solvent accessibility and relative solvent accessibility for wild type and mutant residues [35], [36], [37]. A binary feature was developed to assess whether the wild type or mutant residue was buried and charged [18]. Residues involved in ligand binding were identified from three databases: LigBase, ModBase, and PDBsum [38], [39], [40]. We also developed features to examine the relationship between mutation site and the secondary structure. Thermal factor measures residue rigidity. We calculated thermal factors for the mutated residue as well as the side-chain of the residue. Further, changes in free energy may imply that protein stability is altered when a mutation occurs. We calculated the free energy change between the wild-type and mutant using PoPMuSiC v2.0 [41] and FoldX v3.0 software [42].

#### Neighborhood defined by sequence distance

The sequence neighborhood includes neighbouring residues both upstream and downstream of the mutated residue. We used a 20-dimensional (20-D) vector to record the residue counts by type in the neighborhood [22]. We calculated sequence conservation and physicochemical properties for neighborhood residues. We determined hydrophobic moment [14], residue buriedness, and thermal factors for residues in the sequence neighborhood. Further, we identified ligand binding and functional sites [43], and computed the distance (number of amino acids) from the mutation site to the nearest functional/ligand binding site.

#### Neighborhood defined by Euclidean distance

**Figure 1:**
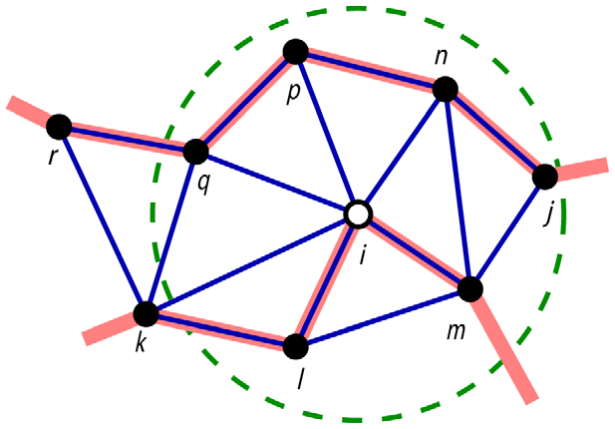
The difference between Euclidean and topological neighborhoods illustrated in two dimensions. The dots are residues and the thick red lines are the protein backbone. The green dotted circle represents the neighborhood defined by Euclidean distance with mutation site *i* (open dot) at the center. The Euclidean neighborhood considers all residues within the circle: *j*, *l*, *m*, *n*, *p*, *q* (solid dots). The blue lines are edges in Delaunay tetrahedra. The topological neighborhood using the same radius cutoff includes *l*, *m*, *n*, *p*, *q*. Residue *j* is not counted because it is not on a Delaunay edge with the mutation site *i*.

The Euclidean neighborhood includes residues located within a sphere of certain radius centred on the mutated residue (Figure 1). We used the coordinates of C_α_ atoms when calculating distances. We counted all neighbouring residues as well as residues by type. Similar to the sequence neighborhood, we calculated sequence conservation, physicochemical properties, residue buriedness, thermal factors, and distance to the nearest functional/ligand binding site for residues in the Euclidean neighborhood. We modelled hydrogen bond and salt bridge interactions using Chimera [44] and counted the number of these interactions in the neighborhood.

#### Neighborhood defined by topological distance

We further considered structural neighborhood based on relative distance, as opposed to the absolute Euclidean distance. The Delaunay tessellation divides the space spanned by a set of points into a collection of non-intersecting tetrahedra in three dimensions (3-D) (triangles in 2-D) [45], [46]. Consequently, Delaunay tetrahedra (DT) define clusters of four nearest neighbours. These clusters are not based on the use of an absolute distance cutoff, and hence are considered a more robust definition of nearest neighbours. Delaunay partitioning of 3-D space has been applied previously to proteins for modelling various aspects of structure and function, including fold recognition [47], mutation effects [48], and blind docking [49]. Our topological neighborhood includes residues that form a Delaunay tetrahedron together with the mutated residue, and are located within a sphere of certain radius centered on the mutated residue (Figure 1). We used the coordinates of C_α_ atoms when calculating distances. We counted all residues as well as residues by type in the topological neighborhood. A unique feature set in this category consists of the number and the type of Delaunay tetrahedra. There are five DT types based on the overlap between backbone and DT edges [47] (Figure S1). We also calculated sequence conservation, physicochemical properties, residue buriedness, and thermal factors for residues in the topological neighborhood.

### TS prediction models

We used logistic regression as the classifier to predict binary response variables: TS versus neutral mutations. The model is formulated as 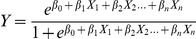, where *Y* represents the posterior probability of an amino acid substitution resulting in a TS mutation, *X_i_* is the *i^th^* predictive feature, and *β_i_* is the corresponding feature coefficient. Given the larger number of neutral mutations in our dataset, the positive (TS) and negative (neutral) training examples were weighted inversely to the number of such examples to mimic a 50-50 mixture of TS and neutral mutations.

We applied a Lasso regularized procedure to select a parsimonious feature set from all studied features. Lasso minimizes the usual sum of squared errors, with a bound on the sum of the absolute values of the coefficients [50]. We implemented Least Angle Regression [51], an efficient Lasso model selection algorithm, to select a subset of features. The subset features were then included in the logistic regression model to predict TS mutations.

### Evaluation of TS prediction models

We evaluated the classification accuracy of TS models by ten-fold cross-validation, where the classifier was built from 90% of the training set and evaluated with the remaining 10%, and the process iterated 10 times. We further applied a leave-one protein-out strategy to evaluate the model performance. Mutations from four proteins were used for building the classifier while mutations from one reserved protein were held out for evaluation. Finally we evaluated our models using independent datasets.

We calculated five measures to assess model performance. By taking 0.5 as the threshold to define TS and neutral mutations, we calculated accuracy (ACC), Matthews correlation coefficient (MCC), and Kullback-Leibler divergence (KL) [52]. ACC and MCC were defined as 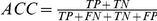 and 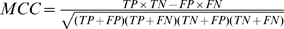, where TP is the number of correctly classified TS mutations, FN is the number of TS mutations predicted to be neutral, TN is the number of correctly classified neutral mutations, and FP is the number of neutral mutations predicted to be TS. Usually, MCC is a better measure than ACC on an unbalanced training set. The Kullback-Leibler divergence was calculated as 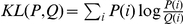, where *i* is TS or neutral, *P(i)* is the predicted probability of *i*, and *Q(i)* is the observed probability of *i*. In addition, two measures were calculated by taking multiple thresholds to define TS and neutral mutations: distribution distance (DD) and area under receiver operator characteristic curve (AUC). The measure DD determines how the predicted TS probability distribution differs from the predicted neutral probability distribution. The formula for DD is the same as that of the Kullback-Leibler divergence, but with the notations modified as follows. The range of the posterior probability (dependent variable) was divided into ten equal intervals. With *i* representing one such interval, *P(i)* is the probability of TS mutants in interval *i*, and *Q(i)* is the probability of neutral mutants in the same interval. A DD value of zero indicates that the predicted neutral and TS distributions are indistinguishable, while large values of DD indicate increased separation between the two distributions.

## Results

### The neighborhood of a mutation site

We investigated three types of neighborhood – sequence, Euclidean, and topological neighborhood. The neighborhood is defined by a distance cutoff. We experimented with different cutoffs using the feature–a 20-D vector of residue counts by type in the neighborhood. A previous study suggested that this neighborhood feature accurately predicted disease-associated mutations [22].

**Figure 2:**
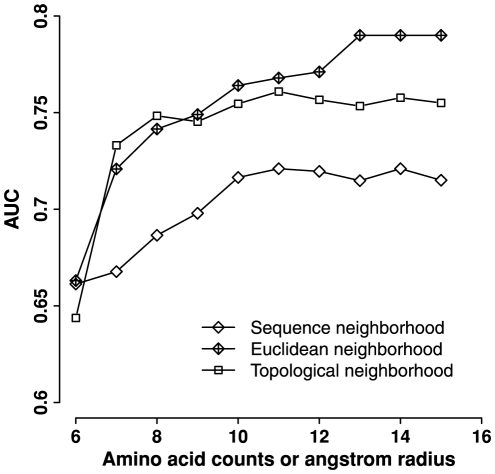
Neighborhood performance at different distance cutoffs. The feature–residue counts by type in the neighborhood–was calculated for sequence, Euclidean, and topological neighborhoods at different distance cutoffs. A classifier was built by using each feature. The AUC value was calculated from a ten-fold cross-validation of the classifier. The x-axis represents the number of residues for the sequence neighborhood or the angstrom radius for the Euclidean and topological neighborhoods.

We built a classifier using the 20-D vector and calculated AUC values from a ten-fold cross-validation of the classifier. For the sequence neighborhood, we varied the number of neighbouring residues from 6 to 15. For both Euclidean and topological neighborhood, we varied the radius of the sphere from 6 Å to 15 Å (Figure 2). We found that the AUC value reached a plateau at 11 residues for the sequence neighborhood. Thus, we included 11 residues upstream and 11 residues downstream of the mutation site as the sequence neighborhood. The AUC value for the Euclidean neighborhood gradually increased with the radius and then reached a plateau at 13 Å. In contrast, the AUC value of the topological neighborhood stayed close to a constant in the radius range of 7–15 Å. This observation is consistent with the definition of Delaunay tetrahedra, which robustly cluster four nearest neighbours together. We chose to use the same cutoff value (13 Å) for the topological and Euclidean neighborhood so that the results are comparable. When comparing the performance of 20-D vectors of the three neighborhoods, we observed that the two structural neighborhoods were more predictive of TS mutations than the linear sequence neighborhood (Figure 2). The optimal cutoff identified by the 20-D vector was applied to the calculation of other neighborhood features.

### Features predictive for TS mutations

The main goal of this study is to elucidate the molecular basis of TS mutations. To this end, the identification of predictive features for TS mutations will enhance our understanding of TS mechanism and help engineer TS mutants for gene functional study. To assess the feature importance in predicting TS mutations, we built 133 individual feature-based classifiers and then performed a ten-fold cross-validation on each classifier. Values of ACC, MCC, KL, DD, and AUC were calculated from cross-validations to rank the 133 features (Table S2, Table S3). The top-20 predictive features for TS mutations are listed in Table 3. We found that neighborhood features dominate the top-ten list, suggesting that neighborhood properties are more predictive than mutation site properties in determining TS mutations. The most predictive feature was the 20-D vector of residue counts by type in the Euclidean neighborhood (AUC = 0.79). The similar 20-D vectors of the topological and sequence neighborhood were highly predictive as well, ranking as the 3^rd^ (AUC = 0.75) and 11^th^ (AUC = 0.72) most predictive features, respectively. The 20-D vector feature performed much better than the total residue counts in the Euclidean neighborhood (AUC = 0.79 versus 0.66) as well as in the topological neighborhood (AUC = 0.75 versus 0.63) (Table S2). This indicates that residue counts by type are more informative for TS mutations than a single count of total residues in the neighborhood.

**Table 3:** The top-20 most predictive features for TS mutations based on AUC values from a ten-fold cross validation.

Rank	Feature name	AUC
1	Residue counts by type in Euclidean neighborhood	0.79
2	Entropy of subfamily	0.76
3	Residue counts by type in topological neighborhood	0.75
4	Entropy of subfamily in topological neighborhood	0.75
5	Relative entropy of subfamily in topological neighborhood	0.75
6	Entropy of subfamily in Euclidean neighborhood	0.74
7	Relative entropy of subfamily in Euclidean neighborhood	0.74
8	Entropy of subfamily in sequence neighborhood	0.74
9	Relative entropy of subfamily in sequence neighborhood	0.73
10	Relative entropy of subfamily	0.73
11	Residue counts by type in sequence neighborhood	0.72
12	Positional hidden Markov model conservation score	0.71
13	Relative solvent accessibility of wild type residue	0.71
14	Side-chain thermal factor	0.70
15	Normalized side-chain thermal factor	0.70
16	Solvent accessibility of wild type residue	0.70
17	Residue thermal factor	0.69
18	Normalized residue thermal factor	0.69
19	Side-chain thermal factor in topological neighborhood	0.69
20	Normalized side-chain thermal factor in topological neighborhood	0.69

Eight top-ten features characterized sequence conservation at the subfamily level. Conservation is quantified by entropy and relative entropy. Not only is the mutated residue highly conserved, the residues in the neighborhood are as well. This suggests that TS mutations tend to occur at conserved protein domains. Our results also indicate that thermal factors are important predictors of TS mutations. Both mutated residues and neighborhood residues tend to locate in rigid regions of a protein. Further, solvent accessibilities of wild-type residues are strong predictors of TS mutations. Residues with low thermal factors and low solvent accessibilities suggest well-defined conformations. These residues can mediate intramolecular interactions that make large contributions to the thermal stability of the protein.

The top-five predictive features by category are listed in Table S4. Consistent with our observation from the top-20 list, we found that neighborhood features are at least as predictive as mutation site features for TS. In fact, top features in both Euclidean and topological neighborhood tend to have higher AUC values than those of top mutation site features. Only sequence neighborhood features are not as predictive as mutation site features.

### Feature independence

**Figure 3:**
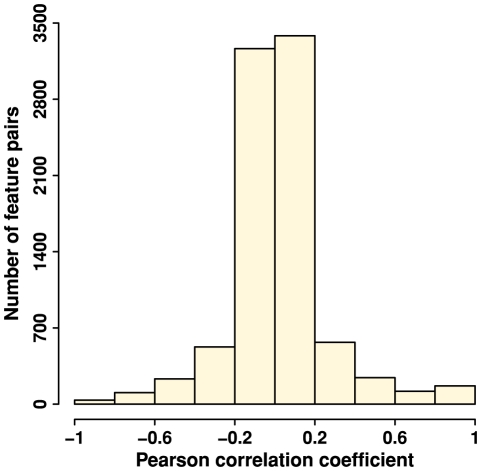
Feature independence quantified by Pearson correlation coefficients. Pearson correlations of feature values on studied mutations were calculated for 8778 all possible feature pairs. A histogram was plotted to illustrate the distribution of Pearson correlation coefficients.

We developed 133 features that describe either the mutation site or its neighborhood. Some features may be redundant. To systematically examine feature independence, we computed Pearson correlation coefficients of feature values on studied mutations for all possible feature pairs, 8778 in total. The correlation coefficients followed a normal distribution centred at 0; more than 75% of the coefficient values were in the range of -0.2 to 0.2 (Figure 3). This suggests that a large majority of features are independent and capture different properties of the mutation site or the neighborhood. The highly positively correlated features (coefficient > 0.8) were either different formats of the same measurement, for example, thermal factor versus normalized thermal factor, or the counterpart features in Euclidean and topological neighborhood. The highly negatively correlated features (coefficient < -0.8) were entropy and relative entropy. This is due to their mathematical formulations.

### TS prediction models

TS mutants are useful in investigating gene functions. They can be efficiently obtained by construction of predicted mutations via targeted mutagenesis. To predict TS mutations derived from single amino acid substitutions, we built TS prediction models using logistic regression. We applied the Lasso regularized procedure [50] to select a subset of all input features to more effectively predict TS mutations. The Lasso is a shrinkage and selection method for linear regression. This procedure eliminates redundant as well as non-informative features from the initial set so that only a parsimonious set of the most informative features are included in the logistic regression model. To this end, a total of eight models were developed to predict TS mutations by using different feature sets: “site features” model, “neighborhood features” model, “sequence neighborhood” model, “Euclidean neighborhood” model, “topological neighborhood” model, “sequence features” model, “structure features” model, and “all features” model (Tables S5, S6, S7, S8, S9, S10, S11, S12).

**Figure 4:**
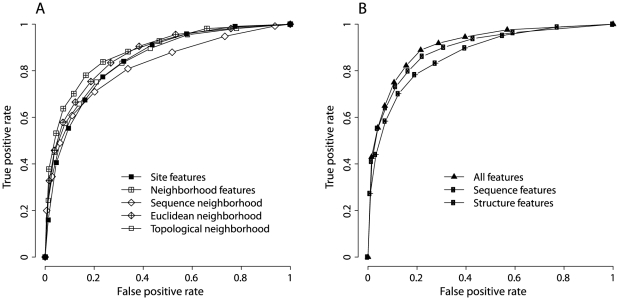
Performance of TS prediction models from a ten-fold cross-validation. TS prediction models were built by applying logistic regression and Lasso regularized procedure. Different models used different sets of features. ROC curves were plotted to evaluate each model. A. TS prediction models using either mutation site features or neighborhood features. B. TS prediction models using all features, sequence features, or structure features.

We evaluated the performance of TS prediction models by plotting receiver operating characteristic (ROC) curves from a ten-fold cross-validation (Figure 4). We also calculated four other performance measures: ACC, MCC, KL, and DD (Table S13). The “site features” model used mutation site features, while the “neighborhood features” model used neighborhood features. We observed that the “neighborhood features” model outperformed the “site features” model in predicting TS mutations (Figure 4A). This result is consistent with the performance of individual features (Table 3) and, again, indicates that neighborhood features are more important than mutation site features in predicting TS mutations. The neighborhood features were further divided into three sets, which served as the input for the “sequence neighborhood” model, the “Euclidean neighborhood” model, and the “topological neighborhood” model. We found the three neighborhood models contain similar numbers of features to that of the “site features” model. However, the performance of the Euclidean and topological neighborhood models is either better than or comparable to that of the “site features” model. Only the “sequence neighborhood” model performs worse than the “site features” model.

Next, we were interested in testing the predictive power of sequence and structure features. The “sequence features” model contained features calculated from sequence information only, while the “structure features” model had features derived from the protein crystal structure. The “all features” model combined sequence and structure features. The performance of the “all features” model was slightly better than that of the “structure features” model, while the “structure features” model performed better than the “sequence features” model (Figure 4B). This suggests that structure-based features have higher predictive power than sequence-based features in differentiating TS mutations from neutral mutations. Nevertheless, the results demonstrated that the “sequence features” model is useful in predicting TS mutations. For a false positive rate of 20%, the “sequence features” model achieved a true positive rate of 78% from a ten-fold cross-validation. Ideally, we use the “all features” model to predict TS mutations (AUC = 0.91). When structural information is absent for a protein, we use the “sequence features” model for broader application (AUC = 0.87).

**Figure 5:**
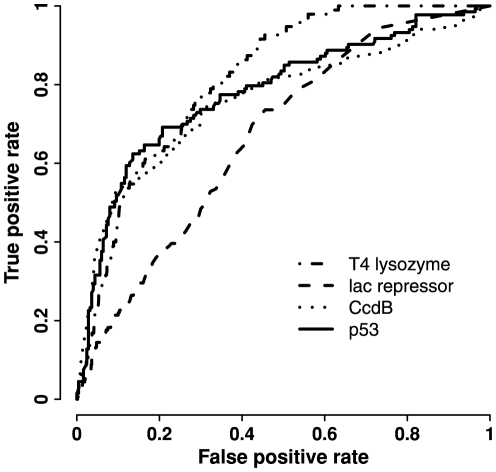
Performance of TS prediction models from leave-one protein-out validation. Mutations from four proteins were used to build the “all features” model by applying logistic regression and Lasso regularized procedure, and mutations from one reserved protein as indicated in the legend were used to evaluate the model by ROC curve.

Our training data include TS and neutral mutations of five proteins (Table 1). We adopted a second strategy termed leave-one protein-out to further evaluate the performance of TS prediction models. Mutations from four proteins were used to build the “all features” model by applying logistic regression and Lasso regularized procedure as described before. Mutations from the remaining one protein were reserved to validate the model. Mutations from T4 lysozyme, *lac* repressor, CcdB, and p53 served as the independent testing set, respectively, because these four proteins have both TS and neutral mutations (Figure 5). Our results showed that AUC values for the four models ranged from 0.67 to 0.82, among which the model by leaving-T4 lysozyme-out achieved the best performance. This result can be explained by the size of training examples, as T4 lysozyme had the smallest number of TS mutations among all proteins.

The “all features” model based on ten-fold cross validation (AUC = 0.91) performed better than the leave-one protein-out “all features” models (AUC = 0.67–0.82). Two reasons may account for the difference. First, more training data was used for building the model based on cross validation than for building the leave-one protein-out models. Second, the leave-one protein-out models were evaluated by different sizes of independent dataset while the other model was evaluated by cross validation. Since leave-one protein-out procedure applied independent dataset to assess TS prediction models, it provides an indication on model performance for real-world applications.

### Validation of TS prediction models with independent datasets

To further assess the performance of TS prediction models, we obtained the mutation data on HIV-1 protease [53], [54]. This independent testing set consists of 14 TS and 110 neutral mutations. The permissive temperature of these mutations was 32°C and the restrictive temperature was 37°C, which are similar to those of our five training proteins. PDB structure of 2BPV_A was used for calculating structure features.

**Figure 6:**
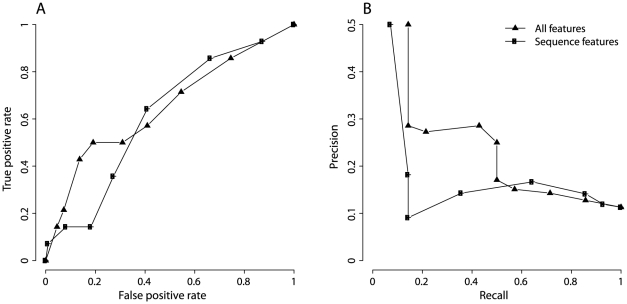
Performance of TS prediction models on an independent testing set. The independent testing set consists of 14 TS and 110 neutral mutations of HIV-1 protease. The performances of “all features” model and “sequence features” model were evaluated by this testing set. A. ROC curves. B. Precision-recall curves.

As shown in Figure 6A, the ROC curve of the “all features” model is biphasic. True positive rates are three-fold of false positive rates when applying thresholds greater than 0.5, while true positive rates are less than two-fold of false positive rates when applying thresholds less than 0.5. Although the AUC value is slightly lower than those of leave-one protein-out (0.65 versus 0.67-0.82), the lower left portion of the ROC curve is comparable with those, suggesting our top-ranked predictions are particularly accurate. We further plotted precision-recall curves to evaluate the performance of the “all features” model (Figure 6B). A 50% precision is reached when 14% of total TS mutations are covered. As thresholds decline, the recall increases but the precision of TS prediction decreases, suggesting that a higher number of TS mutations are recovered at the expense of adding more neutral mutations to the list. When the same independent testing set was used to evaluate the “sequence features” model, an AUC value of 0.61 is reached for the ROC curve and a 50% precision is achieved with a 7% recall (Figure 6). Although the “all features” model outperforms the “sequence features” model, the “sequence features” model has broader application in predicting TS mutations, as structural information is absent for many proteins.

Our goal is to develop a computational method to efficiently obtain TS mutants, not to identify all TS mutations for a given protein. The prediction accuracy of top-ranked TS mutations is much more interesting than the overall accuracy in classifying TS and neutral mutations. Therefore, applying a high threshold to define a small number of TS mutations is the most efficient way to experimentally construct and obtain TS mutants. Both “all features” and “sequence features” models achieved a 50% precision in predicting TS mutations. This is a dramatic enrichment given the fraction of TS mutations among all possible single amino acid substitutions in a protein is in the range of 4–6% [10], [11], [12].

**Figure 7:**
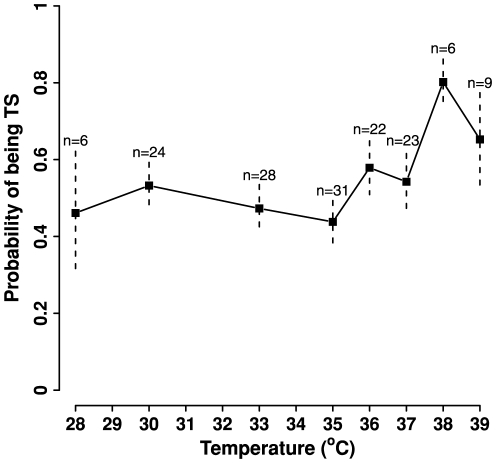
Performance of the “sequence features” model on an independent TS mutant set. A total of 149 TS mutants from 110 yeast genes form the independent testing set. The mutants were grouped based on their restrictive temperatures. The probability of being TS was calculated for each mutant using the “sequence features” model. Average probabilities with standard errors are shown for each group, ranging in size from n = 6 to n = 31.

To further assess the “sequence features” model, we obtained the second independent testing set containing 149 single TS mutants of 110 yeast essential genes [55]. We calculated the probability of these mutants being TS using the “sequence features” model because not all proteins have structural information. As the restrictive temperatures of these mutants vary from 28°C to 39°C, we grouped the mutants based on their restrictive temperatures (Figure 7). We found the “sequence features” model has no predictive power for TS mutants with restrictive temperatures of 37°C or below, because the predicted probability of being TS fluctuates around 0.5. However, the predicted probability of being TS rises to 0.8 and 0.65 for TS mutants with restrictive temperature of 38°C and 39°C, respectively. This is consistent with the fact that all our training mutants were evaluated at the restrictive temperatures of 37°C (Table 1). Therefore, our models would perform best for predicting TS mutants with restrictive temperatures above 37°C.

### Comparison with other methods

Although there has been considerable interest in applying machine learning methods to predict the effects of non-synonymous mutations, the majority of the work focused on deleterious mutations or disease associated mutations [16], [17], [18], [19], [20], [21], [22], [23], [24], [25], [26], [27]. Previous efforts outlined structure- and sequence-based criteria for designing TS mutations of globular proteins [14], [15]. They suggest first the identification of buried sites or ligand binding sites and then random mutation of one site. As these *ad hoc* criteria have no underlying statistical framework, it is infeasible to compare them with our TS prediction models.

**Figure 8:**
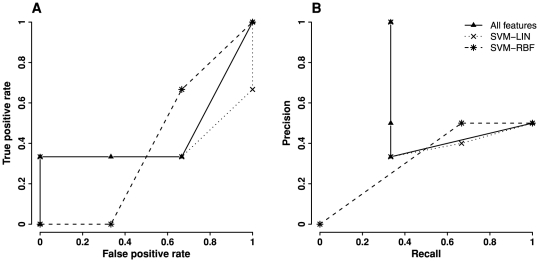
Performance comparison between the “all features” model and an existing method on an independent testing set. The independent testing set consists of 3 TS and 3 neutral mutations of HIV-1 protease. The performances of “all features” model and SVM-LIN/SVM-RBF [28] were evaluated by this testing set. A. ROC curves. B. Precision-recall curves.

A recently published study presented a method to predict TS mutants but the evaluation is based on cross-validation only [28]. The method used support vector machine (SVM) with a smaller training set than ours (75 TS and 130 non-TS). It developed a similar number of structure and sequence features as ours (108 features), most of which are Rosetta relax-derived features. Further, it only considered mutations on buried residues while our method ranks mutations on all residues. To objectively compare the performance of our method with the existing one, we tested them on the independent mutation data of HIV-1 protease. However, only six out of 124 mutations were predicted to have confidence score using the existing method; the rest are either not on buried residues or have no confidence score. Performance comparison based on these six mutations (3 TS and 3 neutral) is shown in Figure 8. The result indicates that our “all features” model either outperforms the existing method or achieves similar performance as the existing one.

Further, based on cross-validation on identical training data, we can compare the performance of our model with other machine learning approaches. We built a SVM classifier to predict TS mutations. SVM classifiers identify a hyperplane that can best separate TS and neutral mutations in a high-dimensional space [56]. Same training data and same feature set were used for SVM as those for the “all features” model. The SVM classifier was trained and built with Matlab interface for the Libsvm package [57]. Different kernel functions were tested, including linear, polynomial, and radial basis function. The best combination of parameters was selected by a grid-search. Based on a ten-fold cross-validation, we obtained the best-performed SVM classifier with polynomial kernel using degree of 3, SVM parameter *γ* of 0.004, and penalty parameter *C* of 0.00011. The AUC of this SVM classifier was 0.91, which is same as that of the “all features” model; both classifiers were evaluated via the identical cross validation procedure. This result demonstrates that our logistic regression models have similar performance as the SVM classifier.

### Comparison of performance measures

Multiple measures can improve the confidence in performance evaluation. We calculated five measures to evaluate the performance of features (Table S2) and TS prediction models (Table S13) from cross-validations. To compare the five measures, we computed the Pearson correlation and mutual information between every pair of the five measures across all 133 individual feature-based classifiers (Table 4). Pearson correlation is only sensitive to a linear relationship between two variables while mutual information is capable of detecting non-linear relationships. We found that AUC and DD had the best concordance according to mutual information and the third best concordance according to the Pearson correlation. Both measures consider average classification performance by taking multiple thresholds to define TS and neutral mutations. MCC also showed consistent performance with AUC and DD, suggesting it is a highly indicative measure with just one threshold.

**Table 4:** Pearson correlation coefficients (top right) and mutual information (bottom left) between measures across 133 individual feature-based classifiers.

	ACC	MCC	AUC	KL	DD
ACC		0.48	0.45	-0.94	0.43
MCC	20.8		0.93	-0.32	0.83
AUC	19.6	25.9		-0.37	0.85
KL	14.4	14.3	13.2		-0.31
DD	29.0	42.3	50.0	17.5	

## Discussion

The main goal of this study is to elucidate the molecular basis of TS mutations and to predict TS mutations derived from single amino acid substitutions. As such, we tested sequence and structure features individually first, and then built eight different TS prediction models. We assembled 133 features to describe property changes occurring at mutation sites and in mutation site neighborhoods. Three neighborhoods were defined based on sequence, Euclidean, and topological distances. Our results highlight the importance of neighborhood features in predicting TS mutations. The top-predictive features suggest that TS mutations tend to occur at buried and rigid regions and tend to locate at conserved domains. This perfectly explains the importance of neighborhood features. Buried residues and the environment of buried residues contribute greatly to protein stability. Thus, TS mutations represent temperature-induced changes in protein stability. The change in protein stability leads to reversible functional changes.

Our study applied three types of distance to define mutation site neighborhood. Comparisons of these neighborhoods as predictors of TS mutations have not been done before. We found that Euclidean and topological neighborhood performed better than the sequence neighborhood. This is due to the use of structural information. Euclidean and topological neighborhoods have similar predictive power, although the performance of the topological neighborhood is more robust in response to different radius cutoffs.

In addition to testing each feature individually to determine their contribution to the TS phenotype, TS prediction models were developed by performing Lasso logistic regression on feature sets. The Lasso regularized procedure was used to exclude redundant and insignificant features. Many features that were top-ranked individually, such as the residue counts by type in the neighborhood, were selected by the Lasso procedure for TS prediction models. Ideally, the “all features” model would be used to predict TS mutations. However, when protein structural information is absent, we could use the “sequence features” model for broader application. Both “all features” and “sequence features” models predicted TS mutations with a 50% precision through test on independent datasets. This is a dramatic enrichment as compared to 4-6% TS mutations out of all possible single amino acid substitutions [10], [11], [12]. Thus, our models allow TS mutants to be systematically constructed with minimal effort.

In summary, our method provides an efficient route to TS mutants for characterizing gene function, and is in principle applicable to proteins in model systems from bacteria to mammalian cell cultures. With the increased number of TS mutations available in the future, our TS perdition models can be further improved and the TS mechanism can be better understood.

## Supporting Information

Figure S1 **Five types of Delaunay tetrahedra based on backbone chain connectivity in three dimensions**. Blue lines are edges in Delaunay tetrahedra and red lines are the protein backbone.
(PDF)

Table S1 **The detailed description of 133 features**. Features were grouped into mutation site features and neighborhood features. The abbreviated feature name is inside the brackets following the full name.
(PDF)

Table S2 **The performance of 133 features.**
(PDF)

Table S3 **The performance of disordered region, helix breaker, and turn breaker defined by alternative methods**. Performance is similar to those in Table S2.
(PDF)

Table S4 **The top-five most predictive features based on AUC values in each of the six categories.**
(PDF)

Table S5 **The “site features” model.**
(PDF)

Table S6 **The “neighborhood features” model.**
(PDF)

Table S7 **The “sequence neighborhood” model.**
(PDF)

Table S8 **The “Euclidean neighborhood” model.**
(PDF)

Table S9 **The “topological neighborhood” model.**
(PDF)

Table S10 **The “all features” model.**
(PDF)

Table S11 **The “sequence features” model.**
(PDF)

Table S12 **The “structure features” model.**
(PDF)

Table S13 **Performance of TS prediction models from a ten-fold cross-validation.**
(PDF)

## References

[1] Hartwell LH, Culotti J, Reid B (1970). Genetic control of the cell-division cycle in yeast. I. Detection of mutants.. Proc Natl Acad Sci U S A.

[2] Gorjanacz M, Klerkx EP, Galy V, Santarella R, Lopez-Iglesias C (2007). Caenorhabditis elegans BAF-1 and its kinase VRK-1 participate directly in post-mitotic nuclear envelope assembly.. Embo J.

[3] Konishi A, de Lange T (2008). Cell cycle control of telomere protection and NHEJ revealed by a ts mutation in the DNA-binding domain of TRF2.. Genes Dev.

[4] Sawicki SG, Sawicki DL, Younker D, Meyer Y, Thiel V (2005). Functional and genetic analysis of coronavirus replicase-transcriptase proteins.. PLoS Pathog.

[5] Razak ZR, Varkonyi RJ, Kulp-McEliece M, Caslini C, Testa JR (2004). p53 differentially inhibits cell growth depending on the mechanism of telomere maintenance.. Mol Cell Biol.

[6] Wang X, Fonseca BD, Tang H, Liu R, Elia A (2008). Re-evaluating the roles of proposed modulators of mammalian target of rapamycin complex 1 (mTORC1) signaling.. J Biol Chem.

[7] Simchen G (1978). Cell cycle mutants.. Annu Rev Genet.

[8] Ben-Aroya S, Coombes C, Kwok T, O'Donnell KA, Boeke JD (2008). Toward a comprehensive temperature-sensitive mutant repository of the essential genes of Saccharomyces cerevisiae.. Mol Cell.

[9] Huang Z, Sucgang RS, Lin YY, Shi X, Boeke JD (2008). Plasmid-chromosome shuffling for non-deletion alleles in yeast.. Nat Methods.

[10] Rennell D, Bouvier SE, Hardy LW, Poteete AR (1991). Systematic mutation of bacteriophage T4 lysozyme.. J Mol Biol.

[11] Suckow J, Markiewicz P, Kleina LG, Miller J, Kisters-Woike B (1996). Genetic studies of the Lac repressor. XV: 4000 single amino acid substitutions and analysis of the resulting phenotypes on the basis of the protein structure.. J Mol Biol.

[12] Shiraishi K, Kato S, Han SY, Liu W, Otsuka K (2004). Isolation of temperature-sensitive p53 mutations from a comprehensive missense mutation library.. J Biol Chem.

[13] Alber T, Sun DP, Nye JA, Muchmore DC, Matthews BW (1987). Temperature-sensitive mutations of bacteriophage T4 lysozyme occur at sites with low mobility and low solvent accessibility in the folded protein.. Biochemistry.

[14] Varadarajan R, Nagarajaram HA, Ramakrishnan C (1996). A procedure for the prediction of temperature-sensitive mutants of a globular protein based solely on the amino acid sequence.. Proc Natl Acad Sci U S A.

[15] Bajaj K, Dewan PC, Chakrabarti P, Goswami D, Barua B (2008). Structural correlates of the temperature sensitive phenotype derived from saturation mutagenesis studies of CcdB.. Biochemistry.

[16] Cai Z, Tsung EF, Marinescu VD, Ramoni MF, Riva A (2004). Bayesian approach to discovering pathogenic SNPs in conserved protein domains.. Hum Mutat.

[17] Dobson RJ, Munroe PB, Caulfield MJ, Saqi MA (2006). Predicting deleterious nsSNPs: an analysis of sequence and structural attributes.. BMC Bioinformatics.

[18] Karchin R, Kelly L, Sali A (2005). Improving functional annotation of non-synonomous SNPs with information theory..

[19] Krishnan VG, Westhead DR (2003). A comparative study of machine-learning methods to predict the effects of single nucleotide polymorphisms on protein function.. Bioinformatics.

[20] Ng PC, Henikoff S (2001). Predicting deleterious amino acid substitutions.. Genome Res.

[21] Ramensky V, Bork P, Sunyaev S (2002). Human non-synonymous SNPs: server and survey.. Nucleic Acids Res.

[22] Ye ZQ, Zhao SQ, Gao G, Liu XQ, Langlois RE (2007). Finding new structural and sequence attributes to predict possible disease association of single amino acid polymorphism (SAP).. Bioinformatics.

[23] Adzhubei IA, Schmidt S, Peshkin L, Ramensky VE, Gerasimova A (2010). A method and server for predicting damaging missense mutations.. Nat Methods.

[24] Bromberg Y, Yachdav G, Rost B (2008). SNAP predicts effect of mutations on protein function.. Bioinformatics.

[25] Chasman D, Adams RM (2001). Predicting the functional consequences of non-synonymous single nucleotide polymorphisms: structure-based assessment of amino acid variation.. J Mol Biol.

[26] Ng PC, Henikoff S (2003). SIFT: Predicting amino acid changes that affect protein function.. Nucleic Acids Res.

[27] Yue P, Melamud E, Moult J (2006). SNPs3D: candidate gene and SNP selection for association studies.. BMC Bioinformatics.

[28] Poultney CS, Butterfoss GL, Gutwein MR, Drew K, Gresham D (2011). Rational design of temperature-sensitive alleles using computational structure prediction.. PLoS One.

[29] Cormack BP, Struhl K (1993). Regional codon randomization: defining a TATA-binding protein surface required for RNA polymerase III transcription.. Science.

[30] Kato S, Han SY, Liu W, Otsuka K, Shibata H (2003). Understanding the function-structure and function-mutation relationships of p53 tumor suppressor protein by high-resolution missense mutation analysis.. Proc Natl Acad Sci U S A.

[31] Berman HM, Westbrook J, Feng Z, Gilliland G, Bhat TN (2000). The Protein Data Bank.. Nucleic Acids Res.

[32] Zamyatin AA (1972). Protein volume in solution.. Prog Biophys Molec Biol.

[33] Grantham R (1974). Amino acid difference formula to help explain protein evolution.. Science.

[34] Ward JJ, Sodhi JS, McGuffin LJ, Buxton BF, Jones DT (2004). Prediction and functional analysis of native disorder in proteins from the three kingdoms of life.. J Mol Biol.

[35] Kabsch W, Sander C (1983). Dictionary of protein secondary structure: pattern recognition of hydrogen-bonded and geometrical features.. Biopolymers.

[36] Eswar N, Eramian D, Webb B, Shen MY, Sali A (2008). Protein structure modeling with MODELLER.. Methods Mol Biol.

[37] Rost B, Sander C (1994). Conservation and prediction of solvent accessibility in protein families.. Proteins.

[38] Laskowski RA (2001). PDBsum: summaries and analyses of PDB structures.. Nucleic Acids Res.

[39] Pieper U, Eswar N, Webb BM, Eramian D, Kelly L (2009). MODBASE, a database of annotated comparative protein structure models and associated resources.. Nucleic Acids Res.

[40] Stuart AC, Ilyin VA, Sali A (2002). LigBase: a database of families of aligned ligand binding sites in known protein sequences and structures.. Bioinformatics.

[41] Dehouck Y, Grosfils A, Folch B, Gilis D, Bogaerts P (2009). Fast and accurate predictions of protein stability changes upon mutations using statistical potentials and neural networks: PoPMuSiC-2.0.. Bioinformatics.

[42] Schymkowitz JW, Rousseau F, Martins IC, Ferkinghoff-Borg J, Stricher F (2005). Prediction of water and metal binding sites and their affinities by using the Fold-X force field.. Proc Natl Acad Sci U S A.

[43] Yip YL, Scheib H, Diemand AV, Gattiker A, Famiglietti LM (2004). The Swiss-Prot variant page and the ModSNP database: a resource for sequence and structure information on human protein variants.. Hum Mutat.

[44] Pettersen EF, Goddard TD, Huang CC, Couch GS, Greenblatt DM (2004). UCSF Chimera - A Visualization System for Exploratory Research and Analysis.. *J Comput Chem*.

[45] Singh RK, Tropsha A, Vaisman II (1996). Delaunay tessellation of proteins: four body nearest-neighbor propensities of amino acid residues.. J Comput Biol.

[46] Preparata FP, Shamos MI (1985). Computational Geometry: An Introduction..

[47] Krishnamoorthy B, Tropsha A (2003). Development of a four-body statistical pseudo-potential to discriminate native from non-native protein conformations.. Bioinformatics.

[48] Masso M, Vaisman II (2007). Accurate prediction of enzyme mutant activity based on a multibody statistical potential.. Bioinformatics.

[49] Aita T, Nishigaki K, Husimi Y (2010). Toward the fast blind docking of a peptide to a target protein by using a four-body statistical pseudo-potential.. Comput Biol Chem.

[50] Tibshirani R (1996). Regression shrinkage and selection via the lasso.. Journal of the Royal Statistical Society Series B.

[51] Efron B, Johnstone I, Hastie T, Tibshirani R (2004). Least angle regression.. The Annals of Statistics.

[52] Kullback S, Leibler RA (1951). On Information and Sufficiency.. The Annals of Mathematical Statistics.

[53] Loeb DD, Swanstrom R, Everitt L, Manchester M, Stamper SE (1989). Complete mutagenesis of the HIV-1 protease.. Nature.

[54] Manchester M, Everitt L, Loeb DD, Hutchison CA, Swanstrom R (1994). Identification of temperature-sensitive mutants of the human immunodeficiency virus type 1 protease through saturation mutagenesis. Amino acid side chain requirements for temperature sensitivity.. J Biol Chem.

[55] Li Z, Vizeacoumar FJ, Bahr S, Li J, Warringer J (2011). Systematic exploration of essential yeast gene function with temperature-sensitive mutants.. Nat Biotechnol.

[56] Schölkopf B, Smola AJ (2002). Learning with Kernels: MIT Press.

[57] LIBSVM website.. http://www.csie.ntu.edu.tw/~cjlin/libsvm/.

