# Supplementary material for: Neighborhood Properties Are Important Determinants of Temperature Sensitive Mutations
Source: PLoS One. 2011 Dec 2;6(12):e28507. doi: 10.1371/journal.pone.0028507 (PMC3229608; doi:10.1371/journal.pone.0028507)
Supplement: Figure S1 — Five types of Delaunay tetrahedra based on backbone chain connectivity in three dimensions. Blue lines are edges in Delaunay tetrahedra and red lines are the protein backbone. (PDF) [file pone.0028507.s001.pdf]

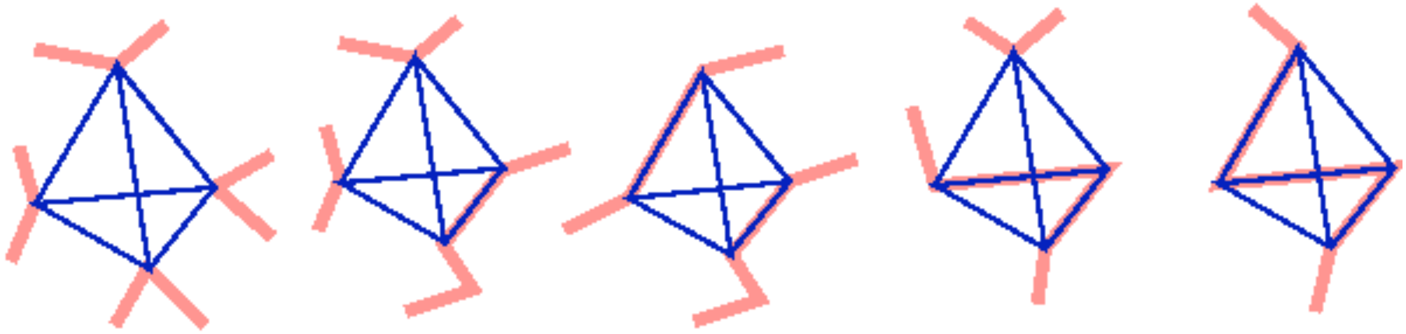

Figure S1 - Five types of Delaunay tetrahedra based on backbone chain connectivity in three dimensions
Blue lines are edges in Delaunay tetrahedra and red lines are the protein backbone.
