# Supplementary material for: Neighborhood Properties Are Important Determinants of Temperature Sensitive Mutations
Source: PLoS One. 2011 Dec 2;6(12):e28507. doi: 10.1371/journal.pone.0028507 (PMC3229608; doi:10.1371/journal.pone.0028507)
Supplement: Table S1 — The detailed description of 133 features. Features were grouped into mutation site features and neighborhood features. The abbreviated feature name is inside the brackets following the full name. (PDF) [file pone.0028507.s002.pdf]

Table S1 - The detailed description of 133 features

Features were grouped into mutation site features and neighborhood features*. The abbreviated feature name is inside the brackets following the full name.

***Mutation site*, sequence features**

1. Entropy of superfamily (EntropySuper): Superfamily alignment was obtained from the SAM-T02 web server [1]. Entropy was calculated as $-\sum_{x=20\ \text{amino acids}} \Pr(x) Log(\Pr(x))$, where *Pr(x)* represents the probability of each amino acid occurring in that position.
2. Relative entropy of superfamily (RelEntropySuper): Entropy was normalized against the distribution of all amino acids in the superfamily alignment: $\sum_{x=20\ \text{amino acids}} \Pr(x) Log(\Pr(x)/\Pr(x_b))$, where *Pr(x)* represents the probability of each amino acid in that position and $Pr(x_b)$ represents the overall probability of occurrence of each amino acid in the superfamily alignment.

3. Entropy of subfamily (EntropySub): Subfamily alignment was constructed manually from the superfamily by including ten orthologs having the highest sequence identity to the query. The entropy formula is same as above.
4. Relative entropy of subfamily (RelEntropySub): The above relative entropy formula was applied to the subfamily alignment.
5. Positional hidden Markov model conservation score (PHC): The score was computed by the formula: $Log(|\Pr(\text{wild}-\text{type}) - \Pr(\text{mutant})|) + Log(\Pr(\text{wild}-\text{type})) + Log(\Pr(\text{most favored})) - Log(\Pr(\text{mutant}))$ [2], where *Pr(wild-type)*, *Pr(mutant)*, *Pr(most favored)* were derived from a hidden Markov model provided by the SAM-T02 web server [1].
6. Hydrophobicity of wild type residue (HydrophobWT): The hydrophobicity of wild-type amino acid was obtained from the following scale: C-100, F-92, I-92, V-87, W-85, M-85, L-85, H-67, Y-62, A-56, G-51, T-46, S-36, R-31, P-31, N-28, Q-26, E-26, D-26, K-0 [3].
7. Hydrophobicity of mutant residue (HydrophobMut): The hydrophobicity of mutant residue was obtained from the above scale.
8. Change in hydrophobicity (HydrophobDiff): The hydrophobicity difference between wild type and mutant residues.
9. Change in volume (VolumeDiff): The size difference between wild type and mutant residues. The sizes of amino acids are as follows: A-88, R-173, N-114, D-111, C-108, Q-144, E-138, G-60, H-153, I-167, L-167, K-168, M-163, F-190, P-112, S-89, T-116, W-227, Y-193, V-140 [4].
10. Change in formal charge (ChargeDiff): The charge difference between wild type and mutant residues.
11. Grantham value (Grantham): The measurement of difference between wild type and mutant residues calculated by considering three properties: composition, polarity, and volume [5].
12. Unusual residue (Unusual): This is a binary variable, equaling 1 if the wild type residue is P or G, and 0 otherwise.
13. Nonpolar wild type residue (NonPolarWT): We classified twenty amino acids into three groups: non-polar (AVFMLGIPWY), polar (STCNQ), and charged (EDKHR). This is a binary variable, equaling 1 if the wild type residue is nonpolar, and 0 otherwise.
14. Polar wild type residue (PolarWT): This is a binary variable, equaling 1 if the wild type residue is polar, and 0 otherwise.
15. Charged wild type residue (ChargedWT): This is a binary variable, equaling 1 if the wild type residue is charged, and 0 otherwise.
16. Nonpolar mutant residue (NonPolarMut): This is a binary variable, equaling 1 if the mutant residue is nonpolar, and 0 otherwise.
17. Polar mutant residue (PolarMut): This is a binary variable, equaling 1 if the mutant residue is polar, and 0 otherwise.
18. Charged mutant residue (ChargedMut): This is a binary variable, equaling 1 if the mutant residue is charged, and 0 otherwise.
19. Nonpolar mutated to charged (NonPolar2Charged): This is a binary variable, equaling 1 if a nonpolar residue is mutated to a charged residue, and 0 otherwise.
20. Nonpolar mutated to polar (NonPolar2Polar): This is a binary variable, equaling 1 if a nonpolar residue is mutated to a polar residue, and 0 otherwise.
21. Nonpolar mutated to nonpolar (NonPolar2NonPolar): This is a binary variable, equaling 1 if a nonpolar residue is mutated to a nonpolar residue, and 0 otherwise.
22. Polar mutated to charged (Polar2Charged): This is a binary variable, equaling 1 if a polar residue is mutated to a charged residue, and 0 otherwise.
23. Polar mutated to polar (Polar2Polar): This is a binary variable, equaling 1 if a polar residue is mutated to a polar residue, and 0 otherwise.
24. Polar mutated to nonpolar (Polar2NonPolar): This is a binary variable, equaling 1 if a polar residue is mutated to a nonpolar residue, and 0 otherwise.
25. Charged mutated to charged (Charged2Charged): This is a binary variable, equaling 1 if a charged residue is mutated to a charged residue, and 0 otherwise.
26. Charged mutated to polar (Charged2Polar): This is a binary variable, equaling 1 if a charged residue is mutated to a polar residue, and 0 otherwise.
27. Charged mutated to nonpolar (Charged2NonPolar): This is a binary variable, equaling 1 if a charged residue is mutated to a nonpolar residue, and 0 otherwise.
28. Disordered region (DisorderRegion): Disopred2 [6] was used to predict intrinsic disordered regions in a protein. Disordered region is a binary variable, equaling 1 if the mutation is located in a disordered region, and 0 otherwise.

***Mutation site,* structure features**

1. Solvent accessibility of wild type residue (SolvAccessWT): DSSP software [7] was used to calculate the feature with the PDB file as input.
2. Relative solvent accessibility of wild type residue (RelSolvAccessWT): The solvent accessibility was normalized by maximum solvent accessibility for each amino acid as follows: C-135, F-197, I-169, V-142, W-227, M-188, L-164, H-184, Y-222, A-106, G-84, T-142, S-130, R-248, P-136, N-157, Q-198, E-194, D-163, K-205 [8].
3. Solvent accessibility of mutant residue (SolvAccessMut): A homology model for the mutant structure was obtained by applying the 'mutate_model' routine in the software MODELLER [9] to the original PDB file. Then the solvent accessibility of mutant residue was calculated by DSSP using the homology model as input.
4. Relative solvent accessibility of mutant residue (RelSolvAccessMut): The solvent accessibility was normalized by maximum solvent accessibility for each amino acid as above.
5. Change in solvent accessibility (SolvAccessDiff): The difference in solvent accessibility between wild type and mutant residues.
6. Change in relative solvent accessibility (RelSolvAccessDiff): The difference in relative solvent accessibility between wild type and mutant residues.
7. Buried and charged wild type residue (BuryWT): A binary variable equaling 1 if the wild type residue is charged and buried (relative solvent accessibility < 16%) and 0 if not.
8. Buried and charged mutant residue (BuryMut): A binary variable equaling 1 if the mutant residue is charged and buried (relative solvent accessibility < 16%) and 0 if not.
9. Ligand binding (IsLigand): This is a binary variable, equaling 1 if the residue is involved in ligand binding, and 0 otherwise. The binding residues were identified through searching three databases: LigBase, ModBase, and PDBsum [10,11,12].
10. Secondary structure (InStruct): This is a binary variable equaling 1 if the wild type residue is in a secondary structure (helix, sheet, or turn) defined in the PDB file, and 0 if not.
11. Helix breaker (HelixBreaker): This is a binary variable, equaling 1 if the wild-type residue is P or G and in a helix as defined in the PDB file, and 0 otherwise.
12. Turn breaker (TurnBreaker): This feature is similar to the helix breaker except the wild type residue is in a turn.
13. Residue thermal factor (Bfactor): The average thermal factor of all atoms in a wild type residue. Atom thermal factors were obtained from the PDB file.
14. Normalized residue thermal factor (normBfactor): Residue thermal factor was normalized by subtracting the mean and dividing by the standard deviation of residue thermal factors in a protein.
15. Side-chain thermal factor (sBfactor): This feature considers only atoms in the side-chain of the wild-type residues.
16. Normalized side-chain thermal factor (snormBfactor): Side-chain thermal factor was normalized in the same way as the residue thermal factor.
17. Free energy by PoPMuSiC (ddGPoPMuSiC): The change in free energy between wild type and mutant residues was calculated by using PoPMuSiC v2.0 webserver [13].
18. Free energy by FoldX (ddGratioFoldX): The change in free energy between wild type and mutant residues divided by the free energy of the wild type residue. Free energy was calculated by using FoldX software [14].

***Neighborhood defined by sequence distance*, sequence features**

1. Residue counts by type in sequence neighborhood (AA20D): A 20-D vector of the counts of neighborhood residues by type.
2. Entropy of superfamily in sequence neighborhood (EntropySuperAA): Average superfamily entropy of neighborhood residues.
3. Relative entropy of superfamily in sequence neighborhood (RelEntropySuperAA): Average superfamily relative entropy of neighborhood residues.
4. Entropy of subfamily in sequence neighborhood (EntropySubAA): Average subfamily entropy of neighborhood residues.
5. Relative entropy of subfamily in sequence neighborhood (RelEntropySubAA): Average subfamily relative entropy of neighborhood residues.
6. Average hydrophobicity of wild type (HydroAvgWT): Average hydrophobicity over a seven-residue window with the wild-type residue at the fourth position.
7. Average hydrophobicity of mutant (HydroAvgMut): Average hydrophobicity over a seven-residue window with the mutant residue at the fourth position.
8. Change in average hydrophobicity (HydroAvgDiff): Average hydrophobicity difference between the wild type and mutant.
9. Nonpolar residues in sequence neighborhood (NonPolarAA): Number of nonpolar neighborhood residues.
10. Polar residues in sequence neighborhood (PolarAA): Number of polar neighborhood residues.
11. Charged residues in sequence neighborhood (ChargedAA): Number of charged neighborhood residues.
12. Residue groups in sequence neighborhood (NPCAA): A 3-D vector of the counts of nonpolar, polar, and charged neighborhood residues.
13. Positive residues in sequence neighborhood (PosAA): Number of positive charged neighborhood residues.
14. Negative residues in sequence neighborhood (NegAA): Number of negative charged neighborhood residues.
15. Charge in sequence neighborhood (NetChargeAA): Net charge of neighborhood residues.
16. Charge groups in sequence neighborhood (PNNAA): A 3-D vector of the counts of positive, negative, and net charge of neighborhood residues.

***Neighborhood defined by sequence distance*, structure features**

1. Hydrophobic moment of wild type (HydroMomentWT): It was calculated over a nine continuous residue window with the wild-type residue at the fifth position j: $\left\{\left[\sum_{n=j-4}^{j+4} H(n)\sin(\delta * n)\right]^2 + \left[\sum_{n=j-4}^{j+4} H(n)\cos(\delta * n)\right]^2\right\}^{1/2}$, where *H(n)* is the hydrophobicity of the residue, phase angle $\delta=100^0$ if the wild-type residue is located in a helix and $\delta=180^0$ if in a sheet. Hydrophobic moment was defined as zero if the wild-type residue was not in a secondary structure. The secondary structure information was from the PDB file.
2. Hydrophobic moment of mutant (HydroMomentMut): As above, but considering the mutant amino acid.
3. Change in hydrophobic moment (HydroMomentDiff): Hydrophobic moment difference between wild type and mutant.
4. Solvent accessibility in sequence neighborhood (SolvAccessAA): Average solvent accessibility of neighborhood residues.
5. Relative solvent accessibility in sequence neighborhood (RelSolvAccessAA): Average relative solvent accessibility of neighborhood residues.
6. Thermal factor in sequence neighborhood (BfactorAA): Average thermal factor of neighborhood residues.
7. Normalized thermal factor in sequence neighborhood (normBfactorAA): Average normalized thermal factor of neighborhood residues.
8. Side-chain thermal factor in sequence neighborhood (sBfactorAA): Average side-chain thermal factor of neighborhood residues.
9. Normalized side-chain thermal factor in sequence neighborhood (snormBfactorAA): Average normalized side-chain thermal factor of neighborhood residues.
10. Sequence distance to functional site (AA2FT): Number of amino acids between the mutation site and the nearest functional site. Functional sites were obtained from the Swiss-Prot database [15].
11. Sequence distance to ligand binding site (AA2Ligand): Number of amino acids between the mutation site and the nearest ligand binding site.
12. Sequence distance to ligand binding or functional site (AA2FTLigand): Number of amino acids between the mutation site and the nearest ligand binding or functional site.

***Neighborhood defined by Euclidean distance*, structure features**

1. Residue counts by type in Euclidean neighborhood (Eucl20D): A 20-D vector of the counts of neighborhood residues by type.
2. Residues in Euclidean neighborhood (EuclContact): Number of neighborhood residues.
3. Entropy of superfamily in Euclidean neighborhood (EntropySuperEucl): Average superfamily entropy of neighborhood residues.
4. Relative entropy of superfamily in Euclidean neighborhood (RelEntropySuperEucl): Average superfamily relative entropy of neighborhood residues.
5. Entropy of subfamily in Euclidean neighborhood (EntropySubEucl): Average subfamily entropy of neighborhood residues.
6. Relative entropy of subfamily in Euclidean neighborhood (RelEntropySubEucl): Average subfamily relative entropy of neighborhood residues.
7. Hydrophobicity in Euclidean neighborhood (HydroAvgEucl): Average hydrophobicity of neighborhood residues.
8. Hydrophobicity of wild type in Euclidean neighborhood (HydroWToverAvgEucl): Ratio of hydrophobicity of wild type residue to average hydrophobicity of neighborhood residues.
9. Hydrophobicity of mutant in Euclidean neighborhood (HydroMutoverAvgEucl): Ratio of hydrophobicity of mutant residue to average hydrophobicity of neighborhood residues.
10. Nonpolar residues in Euclidean neighborhood (NonPolarEucl): Number of nonpolar neighborhood residues.
11. Polar residues in Euclidean neighborhood (PolarEucl): Number of polar neighborhood residues.
12. Charged residues in Euclidean neighborhood (ChargedEucl): Number of charged neighborhood residues.
13. Residue groups in Euclidean neighborhood (NPCEucl): A 3-D vector of the counts of nonpolar, polar, and charged neighborhood residues.
14. Positive residues in Euclidean neighborhood (PosEucl): Number of positive charged neighborhood residues.
15. Negative residues in Euclidean neighborhood (NegEucl): Number of negative charged neighborhood residues.
16. Charge in Euclidean neighborhood (NetChargeEucl): Net charge of neighborhood residues.
17. Charge groups in Euclidean neighborhood (PNNEucl): A 3-D vector of the counts of positive, negative, and net charge of neighborhood residues.
18. Solvent accessibility in Euclidean neighborhood (SolvAccessEucl): Average solvent accessibility of neighborhood residues.
19. Relative solvent accessibility in Euclidean neighborhood (RelSolvAccessEucl): Average relative solvent accessibility of neighborhood residues.
20. Thermal factor in Euclidean neighborhood (BfactorEucl): Average thermal factor of neighborhood residues.
21. Normalized thermal factor in Euclidean neighborhood (normBfactorEucl): Average normalized thermal factor of neighborhood residues.
22. Side-chain thermal factor in Euclidean neighborhood (sBfactorEucl): Average side-chain thermal factor of neighborhood residues.
23. Normalized side-chain thermal factor in Euclidean neighborhood (snormBfactorEucl): Average normalized side-chain thermal factor of neighborhood residues.
24. Euclidean distance to functional site (Eucl2FT): Euclidean distance between the mutation site and the nearest functional site.

25. Euclidean distance to ligand binding site (Eucl2Ligand): Euclidean distance between the mutation site and the nearest ligand binding site.
26. Euclidean distance to ligand binding or functional site (Eucl2FTLigand): Euclidean distance between the mutation site and the nearest ligand binding or functional site.
27. Hydrogen bond (Hbond_6A): Number of hydrogen bonds within 6Å of the mutation site.
28. Salt bridge (SaltBridge_6A): Number of salt bridges within 6Å of the mutation site.
29. Layer of hydrogen bond (Hbond_2layers): Number of hydrogen bonds within two layers of the mutation site.
30. Layer of salt bridge (SaltBridge_2layers): Number of salt bridges within two layers of the mutation site.

***Neighborhood defined by topological distance*, structure features**

1. Residue counts by type in topological neighborhood (DT20D): A 20-D vector of the counts of neighborhood residues by type.
2. Residues in topological neighborhood (DTContact): Number of neighborhood residues.
3. Entropy of superfamily in topological neighborhood (EntropySuperDT): Average superfamily entropy of neighborhood residues.
4. Relative entropy of superfamily in topological neighborhood (RelEntropySuperDT): Average superfamily relative entropy of neighborhood residues.
5. Entropy of subfamily in topological neighborhood (EntropySubDT): Average subfamily entropy of neighborhood residues.
6. Relative entropy of subfamily in topological neighborhood (RelEntropySubDT): Average subfamily relative entropy of neighborhood residues.
7. Hydrophobicity in topological neighborhood (HydroAvgDT): Average hydrophobicity of neighborhood residues.
8. Hydrophobicity of wild type in topological neighborhood (HydroWToverAvgDT): Ratio of hydrophobicity of wild type residue to average hydrophobicity of neighborhood residues.
9. Hydrophobicity of mutant in topological neighborhood (HydroMutoverAvgDT): Ratio of hydrophobicity of mutant residue to average hydrophobicity of neighborhood residues.
10. Nonpolar residues in topological neighborhood (NonPolarDT): Number of nonpolar neighborhood residues.
11. Polar residues in topological neighborhood (PolarDT): Number of polar neighborhood residues.
12. Charged residues in topological neighborhood (ChargedDT): Number of charged neighborhood residues.
13. Residue groups in topological neighborhood (NPCDT): A 3-D vector of the counts of nonpolar, polar, and charged neighborhood residues.
14. Positive residues in topological neighborhood (PosDT): Number of positive charged neighborhood residues.
15. Negative residues in topological neighborhood (NegDT): Number of negative charged neighborhood residues.
16. Charge in topological neighborhood (NetChargeDT): Net charge of neighborhood residues.
17. Charge groups in topological neighborhood (PNNDT): A 3-D vector of the counts of positive, negative, and net charge of neighborhood residues.
18. Solvent accessibility in topological neighborhood (SolvAccessDT): Average solvent accessibility of neighborhood residues.
19. Relative solvent accessibility in topological neighborhood (RelSolvAccessDT): Average relative solvent accessibility of neighborhood residues.
20. Thermal factor in topological neighborhood (BfactorDT): Average thermal factor of neighborhood residues.
21. Normalized thermal factor in topological neighborhood (normBfactorDT): Average normalized thermal factor of neighborhood residues.
22. Side-chain thermal factor in topological neighborhood (sBfactorDT): Average side-chain thermal factor of neighborhood residues.
23. Normalized side-chain thermal factor in topological neighborhood (snormBfactorDT): Average normalized side-chain thermal factor of neighborhood residues.
24. DT count (DTcount): Number of Delaunay tetrahedra within 10Å of mutation site.
25. DT type0 count (DTcountType0): Number of Delaunay tetrahedra type0 within 10Å of mutation site.
26. DT type1 count (DTcountType1): Number of Delaunay tetrahedra type1 within 10Å of mutation site.
27. DT type2 count (DTcountType2): Number of Delaunay tetrahedra type2 within 10Å of mutation site.
28. DT type3 count (DTcountType3): Number of Delaunay tetrahedra type3 within 10Å of mutation site.
29. DT type4 count (DTcountType4): Number of Delaunay tetrahedra type4 within 10Å of mutation site.

* Sequence neighborhood includes 11 residues upstream and 11 residues downstream of the mutation site except for features HydroAvgWT, HydroAvgMut, HydroAvgDiff, HydroMomentWT, HydroMomentMut, and HydroMomentDiff that were originally defined in the reference [3].
Euclidean neighborhood includes residues located within a sphere of 13Å centered on the mutation site except for features Hbond_6A and SaltBridge_6A. This is because non-covalent interactions occur locally.
Topological neighborhood includes residues that form a Delaunay tetrahedron together with the mutated residue, and are located within a sphere of 13Å centered on the mutation site except for features DTcount, DTcountType0-4 that were originally defined in the reference [16].

REFERENCES

1. Karplus K, Karchin R, Draper J, Casper J, Mandel-Gutfreund Y, et al. (2003) Combining local-structure, fold-recognition, and new fold methods for protein structure prediction. Proteins 53 Suppl 6: 491-496.
2. Karchin R, Kelly L, Sali A (2005) Improving functional annotation of non-synonomous SNPs with information theory. Pac Symp Biocomput: 397-408.
3. Varadarajan R, Nagarajaram HA, Ramakrishnan C (1996) A procedure for the prediction of temperature-sensitive mutants of a globular protein based solely on the amino acid sequence. Proc Natl Acad Sci U S A 93: 13908-13913.
4. Zamyatin AA (1972) Protein volume in solution. Prog Biophys Molec Biol 24: 107-123.
5. Grantham R (1974) Amino acid difference formula to help explain protein evolution. Science 185: 862-864.
6. Ward JJ, Sodhi JS, McGuffin LJ, Buxton BF, Jones DT (2004) Prediction and functional analysis of native disorder in proteins from the three kingdoms of life. J Mol Biol 337: 635-645.
7. Kabsch W, Sander C (1983) Dictionary of protein secondary structure: pattern recognition of hydrogen-bonded and geometrical features. Biopolymers 22: 2577-2637.
8. Rost B, Sander C (1994) Conservation and prediction of solvent accessibility in protein families. Proteins 20: 216-226.
9. Sali A, Blundell TL (1993) Comparative protein modelling by satisfaction of spatial restraints. J Mol Biol 234: 779-815.
10. Laskowski RA (2001) PDBsum: summaries and analyses of PDB structures. Nucleic Acids Res 29: 221-222.
11. Pieper U, Eswar N, Webb BM, Eramian D, Kelly L, et al. (2009) MODBASE, a database of annotated comparative protein structure models and associated resources. Nucleic Acids Res 37: D347-354.
12. Stuart AC, Ilyin VA, Sali A (2002) LigBase: a database of families of aligned ligand binding sites in known protein sequences and structures. Bioinformatics 18: 200-201.
13. Dehouck Y, Grosfils A, Folch B, Gilis D, Bogaerts P, et al. (2009) Fast and accurate predictions of protein stability changes upon mutations using statistical potentials and neural networks: PoPMuSiC-2.0. Bioinformatics 25: 2537-2543.
14. Schymkowitz JW, Rousseau F, Martins IC, Ferkinghoff-Borg J, Stricher F, et al. (2005) Prediction of water and metal binding sites and their affinities by using the Fold-X force field. Proc Natl Acad Sci U S A 102: 10147-10152.
15. Yip YL, Scheib H, Diemand AV, Gattiker A, Famiglietti LM, et al. (2004) The Swiss-Prot variant page and the ModSNP database: a resource for sequence and structure information on human protein variants. Hum Mutat 23: 464-470.
16. Deutsch C, Krishnamoorthy B (2007) Four-body scoring function for mutagenesis. Bioinformatics 23: 3009-3015.
