# Supplementary material for: Neighborhood Properties Are Important Determinants of Temperature Sensitive Mutations
Source: PLoS One. 2011 Dec 2;6(12):e28507. doi: 10.1371/journal.pone.0028507 (PMC3229608; doi:10.1371/journal.pone.0028507)
Supplement: Table S2 — The performance of 133 features. (PDF) [file pone.0028507.s003.pdf]

Table S2 - The performance of 133 features

Feature	Feature evaluation*				
	ACC	MCC	AUC	KL	DD
***Mutation site*, sequence features**					
EntropySub	0.73	0.32	0.76	0.21	0.30
EntropySuper	0.59	0.16	0.66	0.47	0.08
RelEntropySub	0.70	0.24	0.73	0.24	0.18
RelEntropySuper	0.59	0.16	0.66	0.48	0.07
PHC	0.68	0.22	0.71	0.26	0.13
HydrophobWT	0.60	0.11	0.60	0.39	0.03
HydrophobMut	0.54	0.07	0.55	0.57	0.01
HydrophobDiff	0.59	0.10	0.61	0.42	0.04
VolumeDiff	0.54	0.05	0.55	0.52	0.01
ChargeDiff	0.41	0.01	0.51	1.02	0.00
Grantham	0.52	0.01	0.50	0.57	0.00
Unusual	0.82	0.04	0.48	0.01	0.00
NonPolarWT	0.54	0.13	0.58	0.63	0.04
PolarWT	0.33	0.09	0.52	1.57	0.02
ChargedWT	0.33	0.06	0.51	1.50	0.01
NonPolarMut	0.52	-0.04	0.45	0.50	0.00
PolarMut	0.48	-0.02	0.48	0.68	0.00
ChargedMut	0.56	-0.02	0.48	0.41	0.00
NonPolar2Charged	0.80	0.09	0.52	0.00	0.02
NonPolar2Polar	0.78	0.04	0.48	0.00	0.00
NonPolar2NonPolar	0.72	0.05	0.51	0.07	0.00
Polar2Charged	0.19	0.06	0.49	2.48	0.01
Polar2Polar	0.17	0.04	0.48	2.60	0.01
Polar2NonPolar	0.22	0.04	0.49	2.22	0.01
Charged2Charged	0.18	0.04	0.47	2.53	0.00
Charged2Polar	0.24	-0.01	0.47	1.98	0.00
Charged2NonPolar	0.22	0.03	0.50	2.14	0.00
DisorderRegion	0.50	-0.02	0.47	0.60	0.00
***Mutation site*, structure features**					
SolvAccessWT	0.58	0.19	0.70	0.53	0.11
SolvAccessMut	0.55	0.16	0.67	0.63	0.09
SolvAccessDiff	0.50	0.02	0.51	0.65	0.00
RelSolvAccessWT	0.61	0.19	0.71	0.44	0.12
RelSolvAccessMut	0.55	0.16	0.67	0.63	0.09
RelSolvAccessDiff	0.50	-0.01	0.51	0.62	0.00
BuryWT	0.87	0.08	0.48	0.10	0.01
BuryMut	0.83	0.11	0.52	0.00	0.02
IsLigand	0.20	0.02	0.48	2.33	0.00
InStruct	0.71	-0.01	0.48	0.05	0.00
HelixBreaker	0.20	-0.03	0.47	2.23	0.01
TurnBreaker	0.57	-0.03	0.47	0.37	0.00
Bfactor	0.57	0.16	0.69	0.55	0.13
normBfactor	0.57	0.16	0.69	0.55	0.11
sBfactor	0.62	0.20	0.70	0.44	0.17
snormBfactor	0.62	0.20	0.70	0.43	0.15
ddGPoPMuSiC	0.68	0.18	0.67	0.22	0.10
ddGratioFoldX	0.77	0.16	0.65	0.04	0.08
***Neighborhood defined by sequence distance*, sequence features**					
AA20D	0.67	0.22	0.72	0.29	0.17
EntropySubAA	0.68	0.25	0.74	0.30	0.26
EntropySuperAA	0.48	0.11	0.56	0.83	0.05
RelEntropySubAA	0.67	0.25	0.73	0.31	0.23
RelEntropySuperAA	0.48	0.12	0.56	0.84	0.05
HydroAvgWT	0.56	0.10	0.59	0.52	0.02

HydroAvgMut	0.51	0.03	0.52	0.61	0.00
HydroAvgDiff	0.59	0.11	0.61	0.41	0.04
NonPolarAA	0.55	0.07	0.59	0.52	0.03
PolarAA	0.57	0.07	0.55	0.46	0.01
ChargedAA	0.57	0.06	0.54	0.46	0.01
NPCAA	0.63	0.11	0.61	0.30	0.04
PosAA	0.49	0.02	0.53	0.68	0.00
NegAA	0.56	0.04	0.51	0.45	0.00
NetChargeAA	0.50	-0.04	0.48	0.56	0.00
PNNAA	0.54	0.06	0.54	0.53	0.01
***Neighborhood defined by sequence distance*, structure features**					
HydroMomentWT	0.55	0.02	0.52	0.46	0.00
HydroMomentMut	0.49	-0.04	0.46	0.60	0.00
HydroMomentDiff	0.69	0.10	0.59	0.14	0.03
SolvAccessAA	0.56	0.05	0.58	0.48	0.05
RelSolvAccessAA	0.57	0.06	0.58	0.45	0.03
BfactorAA	0.51	0.10	0.63	0.72	0.06
normBfactorAA	0.51	0.10	0.63	0.71	0.06
sBfactorAA	0.52	0.11	0.64	0.67	0.06
snormBfactorAA	0.52	0.11	0.64	0.67	0.06
AA2FT	0.71	0.05	0.57	0.08	0.14
AA2Ligand	0.63	0.02	0.54	0.23	0.15
AA2FTLigand	0.63	0.04	0.55	0.24	0.12
***Neighborhood defined by Euclidean distance*, structure features**					
Eucl20D	0.75	0.32	0.79	0.17	0.32
EuclContact	0.64	0.16	0.66	0.34	0.07
EntropySubEucl	0.68	0.25	0.74	0.30	0.29
EntropySuperEucl	0.50	0.14	0.57	0.81	0.07
RelEntropySubEucl	0.69	0.26	0.74	0.27	0.26
RelEntropySuperEucl	0.50	0.13	0.57	0.81	0.07
HydroAvgEucl	0.57	0.11	0.59	0.49	0.03
HydroWToverAvgEucl	0.60	0.10	0.58	0.38	0.02
HydroMutoverAvgEucl	0.54	0.07	0.57	0.57	0.02
NonPolarEucl	0.64	0.15	0.64	0.32	0.06
PolarEucl	0.57	0.03	0.54	0.42	0.00
ChargedEucl	0.60	0.14	0.62	0.42	0.06
NPCEucl	0.63	0.17	0.66	0.36	0.08
PosEucl	0.65	0.15	0.62	0.29	0.08
NegEucl	0.61	0.07	0.55	0.32	0.01
NetChargeEucl	0.55	0.04	0.56	0.48	0.04
PNNEucl	0.64	0.14	0.63	0.30	0.08
SolvAccessEucl	0.49	0.03	0.51	0.70	0.00
RelSolvAccessEucl	0.50	0.03	0.53	0.66	0.00
BfactorEucl	0.52	0.11	0.67	0.68	0.10
normBfactorEucl	0.52	0.11	0.67	0.68	0.10
sBfactorEucl	0.52	0.12	0.68	0.68	0.11
snormBfactorEucl	0.52	0.12	0.68	0.68	0.11
Eucl2FT	0.66	0.10	0.58	0.23	0.15
Eucl2Ligand	0.51	-0.01	0.52	0.59	0.00
Eucl2FTLigand	0.51	0.00	0.52	0.58	0.00
Hbond_6A	0.59	0.07	0.59	0.39	0.13
SaltBridge_6A	0.53	-0.03	0.47	0.48	0.00
Hbond_2layers	0.55	0.12	0.61	0.57	0.04
SaltBridge_2layers	0.49	-0.03	0.47	0.60	0.00
***Neighborhood defined by topological distance*, structure features**					
DT20D	0.71	0.27	0.75	0.23	0.19

DTContact	0.60	0.12	0.63	0.41	0.05
EntropySubDT	0.68	0.26	0.75	0.31	0.27
EntropySuperDT	0.53	0.13	0.60	0.68	0.06
RelEntropySubDT	0.68	0.25	0.75	0.29	0.26
RelEntropySuperDT	0.51	0.13	0.59	0.73	0.05
HydroAvgDT	0.63	0.17	0.66	0.35	0.08
HydroWToverAvgDT	0.56	0.07	0.55	0.49	0.01
HydroMutoverAvgDT	0.53	0.09	0.60	0.60	0.02
NonPolarDT	0.63	0.16	0.65	0.36	0.07
PolarDT	0.56	0.04	0.56	0.46	0.01
ChargedDT	0.51	0.01	0.51	0.61	0.00
NPCDT	0.64	0.17	0.65	0.32	0.08
PosDT	0.51	-0.02	0.48	0.57	0.00
NegDT	0.46	0.05	0.53	0.86	0.01
NetChargeDT	0.53	0.01	0.52	0.53	0.00
PNNDT	0.46	0.05	0.53	0.85	0.01
SolvAccessDT	0.62	0.16	0.65	0.40	0.07
RelSolvAccessDT	0.61	0.16	0.66	0.42	0.08
BfactorDT	0.53	0.13	0.68	0.66	0.11
normBfactorDT	0.53	0.13	0.68	0.67	0.11
sBfactorDT	0.54	0.14	0.69	0.65	0.15
snormBfactorDT	0.54	0.14	0.69	0.65	0.14
DTcount	0.65	0.17	0.66	0.32	0.07
DTcountType0	0.70	0.15	0.62	0.17	0.05
DTcountType1	0.61	0.10	0.59	0.36	0.02
DTcountType2	0.57	0.10	0.59	0.50	0.05
DTcountType3	0.64	0.14	0.62	0.32	0.07
DTcountType4	0.58	0.12	0.60	0.46	0.06

* ACC = accuracy, MCC = Matthews correlation coefficient, AUC = area under the curve, KL = Kullback-Leibler divergence, DD = distribution distance. These values were calculated from a ten-fold cross-validation of each feature.
