# Supplementary material for: Neighborhood Properties Are Important Determinants of Temperature Sensitive Mutations
Source: PLoS One. 2011 Dec 2;6(12):e28507. doi: 10.1371/journal.pone.0028507 (PMC3229608; doi:10.1371/journal.pone.0028507)
Supplement: Table S3 — The performance of disordered region, helix breaker, and turn breaker defined by alternative methods. Performance is similar to those in Table S2. (PDF) [file pone.0028507.s004.pdf]

Table S3 - The performance of disordered region, helix breaker, and turn breaker defined by alternative methods

Performance is similar to those in Table S2.

Feature	Feature evaluation*				
	ACC	MCC	AUC	KL	DD
Disordered region: residues not in PDB-defined helix, sheet, or turn	0.43	-0.01	0.48	1.14	0.00
Disordered region: FoldIndex [1]	0.21	0.02	0.49	2.23	0.00
Helix breaker: helix is defined by DSSP [2]	0.38	-0.03	0.48	1.11	0.00
Turn breaker: turn is defined by DSSP [2]	0.58	-0.03	0.48	0.32	0.00

* ACC = accuracy, MCC = Matthews correlation coefficient, AUC = area under the curve, KL = Kullback-Leibler divergence, DD = distribution distance. These values were calculated from a ten-fold cross-validation of each feature.

REFERENCES

1. Prilusky J, Felder CE, Zeev-Ben-Mordehai T, Rydberg EH, Man O, et al. (2005) FoldIndex: a simple tool to predict whether a given protein sequence is intrinsically unfolded. Bioinformatics 21: 3435-3438.
2. Kabsch W, Sander C (1983) Dictionary of protein secondary structure: pattern recognition of hydrogen-bonded and geometrical features. Biopolymers 22: 2577-2637.
