# Supplementary material for: Neighborhood Properties Are Important Determinants of Temperature Sensitive Mutations
Source: PLoS One. 2011 Dec 2;6(12):e28507. doi: 10.1371/journal.pone.0028507 (PMC3229608; doi:10.1371/journal.pone.0028507)
Supplement: Table S4 — The top-five most predictive features based on AUC values in each of the six categories. (PDF) [file pone.0028507.s005.pdf]

Table S4 - The top-five most predictive features based on AUC values in each of the six categories

Feature	Feature evaluation*				
	ACC	MCC	AUC	KL	DD
***Mutation site*, sequence features**					
EntropySub	0.73	0.32	0.76	0.21	0.30
RelEntropySub	0.70	0.24	0.73	0.24	0.18
PHC	0.68	0.22	0.71	0.26	0.13
EntropySuper	0.59	0.16	0.66	0.47	0.08
RelEntropySuper	0.59	0.16	0.66	0.48	0.07
***Mutation site*, structure features**					
RelSolvAccessWT	0.61	0.19	0.71	0.44	0.12
sBfactor	0.62	0.20	0.70	0.44	0.17
snormBfactor	0.62	0.20	0.70	0.43	0.15
SolvAccessWT	0.58	0.19	0.70	0.53	0.11
Bfactor	0.57	0.16	0.69	0.55	0.13
***Neighborhood defined by sequence distance*, sequence features**					
EntropySubAA	0.68	0.25	0.74	0.30	0.26
RelEntropySubAA	0.67	0.25	0.73	0.31	0.23
AA20D	0.67	0.22	0.72	0.29	0.17
NPCAA	0.63	0.11	0.61	0.30	0.04
HydroAvgDiff	0.59	0.11	0.61	0.41	0.04
***Neighborhood defined by sequence distance*, structure features**					
sBfactorAA	0.52	0.11	0.64	0.67	0.06
snormBfactorAA	0.52	0.11	0.64	0.67	0.06
normBfactorAA	0.51	0.10	0.63	0.71	0.06
BfactorAA	0.51	0.10	0.63	0.72	0.06
HydroMomentDiff	0.69	0.10	0.59	0.14	0.03
***Neighborhood defined by Euclidean distance*, structure features**					
Eucl20D	0.75	0.32	0.79	0.17	0.32
EntropySubEucl	0.68	0.25	0.74	0.30	0.29
RelEntropySubEucl	0.69	0.26	0.74	0.27	0.26
snormBfactorEucl	0.52	0.12	0.68	0.68	0.11
sBfactorEucl	0.52	0.12	0.68	0.68	0.11
***Neighborhood defined by topological distance*, structure features**					
DT20D	0.71	0.27	0.75	0.23	0.19
EntropySubDT	0.68	0.26	0.75	0.31	0.27
RelEntropySubDT	0.68	0.25	0.75	0.29	0.26
sBfactorDT	0.54	0.14	0.69	0.65	0.15
snormBfactorDT	0.54	0.14	0.69	0.65	0.14

* ACC = accuracy, MCC = Matthews correlation coefficient, AUC = area under the curve, KL = Kullback-Leibler divergence, DD = distribution distance. These values were calculated from a ten-fold cross-validation of each feature.
