# Supplementary material for: Neighborhood Properties Are Important Determinants of Temperature Sensitive Mutations
Source: PLoS One. 2011 Dec 2;6(12):e28507. doi: 10.1371/journal.pone.0028507 (PMC3229608; doi:10.1371/journal.pone.0028507)
Supplement: Table S5 — The “site features” model. (PDF) [file pone.0028507.s006.pdf]

Table S5 - The "site features" model

Feature	Estimate
(Intercept)	-0.682
EntropySub	-3.412
EntropySuper	2.084
RelEntropySub	1.377
PHC	0.504
HydrophobMut	-0.009
HydrophobDiff	-0.002
ChargeDiff	-0.137
Unusual	0.020
NonPolarMut	0.039
NonPolar2Charged	0.010
Polar2Charged	-0.905
Polar2Polar	-0.173
Charged2Charged	-0.444
DisorderRegion	0.548
SolvAccessDiff	-0.003
RelSolvAccessWT	-2.077
BuryWT	0.343
BuryMut	0.311
IsLigand	-0.281
HelixBreaker	-0.750
sBfactor	-0.005
snormBfactor	-0.002
ddGPoPMuSiC	0.167
ddGratioFoldX	0.635
