# Supplementary material for: Neighborhood Properties Are Important Determinants of Temperature Sensitive Mutations
Source: PLoS One. 2011 Dec 2;6(12):e28507. doi: 10.1371/journal.pone.0028507 (PMC3229608; doi:10.1371/journal.pone.0028507)
Supplement: Table S6 — The “neighborhood features” model. (PDF) [file pone.0028507.s007.pdf]

Table S6 - The “neighborhood features” model

Feature	Estimate
(Intercept)	-9.486
AA20D_A	0.041
AA20D_C	0.149
AA20D_D	-0.130
AA20D_F	-0.002
AA20D_G	-0.101
AA20D_H	-0.125
AA20D_I	0.140
AA20D_K	-0.004
AA20D_L	-0.150
AA20D_M	-0.001
AA20D_P	-0.339
AA20D_Q	-0.062
AA20D_S	-0.214
AA20D_T	-0.188
AA20D_V	-0.078
AA20D_W	0.242
AA20D_Y	0.008
RelEntropySubAA	2.672
RelEntropySuperAA	-1.400
HydroAvgWT	-0.003
HydroAvgDiff	0.057
PolarAA	-0.015
NonpolarAA	-0.051
ChargedAA	-0.021
NegAA	0.070
AA2Ligand	-0.032
AA2FTLigand	0.039
HydroMomentMut	-0.005
HydroMomentDiff	-0.005
SolvAccessAA	-0.006
RelSolvAccessAA	1.995
sBfactorAA	0.013
snormBfactorAA	0.013
Eucl20D_ALA	0.086
Eucl20D_ARG	0.023
Eucl20D_ASN	-0.142
Eucl20D_ASP	0.054
Eucl20D_CYS	-0.169
Eucl20D_GLN	0.050
Eucl20D_GLU	-0.123
Eucl20D_GLY	0.114
Eucl20D_ILE	0.218
Eucl20D_LEU	-0.205
Eucl20D_LYS	0.113
Eucl20D_MET	0.237
Eucl20D_PHE	0.091
Eucl20D_PRO	0.124
Eucl20D_THR	0.360
Eucl20D_TRP	-0.307
Eucl20D_TYR	-0.154
Eucl20D_VAL	-0.057
RelEntropySubEucl	4.342
RelEntropySuperEucl	-1.481
HydroAvgEucl	-0.008
HydroWToverAvgEucl	0.573
HydroMutoverAvgEucl	-3.332

PosEucl	0.094
SolvAccessEucl	-0.013
RelSolvAccessEucl	8.019
BfactorEucl	0.005
Eucl2FT	0.050
Eucl2Ligand	-0.035
Eucl2FTLigand	0.020
Hbond_6A	-0.043
SaltBridge_6A	0.120
Hbond_2layers	0.007
SaltBridge_2layers	-0.256
DT20D_A	-0.069
DT20D_C	-0.843
DT20D_D	0.192
DT20D_F	-0.061
DT20D_G	0.043
DT20D_H	-0.005
DT20D_I	0.083
DT20D_K	0.421
DT20D_L	-0.070
DT20D_M	-0.060
DT20D_N	-0.149
DT20D_P	0.191
DT20D_Q	0.318
DT20D_R	0.140
DT20D_S	0.147
DT20D_T	0.065
DT20D_V	-0.295
DT20D_Y	-0.005
EntropySuperDT	-5.188
RelEntropySubDT	2.862
RelEntropySuperDT	-8.278
HydroAvgDT	0.147
HydroMutoverAvgDT	2.195
NonPolarDT	-0.029
ChargedDT	0.226
SolvAccessDT	-0.058
RelSolvAccessDT	6.982
BfactorDT	0.004
DTcountType0	0.073
DTcountType1	0.120
DTcountType2	0.148
DTcountType3	-0.006
