# Supplementary material for: Neighborhood Properties Are Important Determinants of Temperature Sensitive Mutations
Source: PLoS One. 2011 Dec 2;6(12):e28507. doi: 10.1371/journal.pone.0028507 (PMC3229608; doi:10.1371/journal.pone.0028507)
Supplement: Table S7 — The “sequence neighborhood” model. (PDF) [file pone.0028507.s008.pdf]

Table S7 - The “sequence neighborhood” model

Feature	Estimate
(Intercept)	-1.578
AA20D_A	0.003
AA20D_C	0.027
AA20D_E	-0.225
AA20D_F	-0.048
AA20D_G	0.162
AA20D_H	-0.226
AA20D_I	0.088
AA20D_L	-0.356
AA20D_M	0.003
AA20D_P	-0.088
AA20D_S	-0.063
AA20D_V	-0.235
AA20D_W	-0.432
EntropySubAA	-2.836
RelEntropySubAA	4.244
RelEntropySuperAA	-4.973
HydroAvgWT	0.025
HydroAvgDiff	-0.046
PolarAA	-0.013
AA2FT	0.007
AA2FTLigand	0.001
HydroMomentDiff	-0.010
RelSolvAccessAA	3.181
