# Supplementary material for: Neighborhood Properties Are Important Determinants of Temperature Sensitive Mutations
Source: PLoS One. 2011 Dec 2;6(12):e28507. doi: 10.1371/journal.pone.0028507 (PMC3229608; doi:10.1371/journal.pone.0028507)
Supplement: Table S8 — The “Euclidean neighborhood” model. (PDF) [file pone.0028507.s009.pdf]

Table S8 - The “Euclidean neighborhood” model

Feature	Estimate
(Intercept)	-1.320
Eucl20D_ALA	0.085
Eucl20D_ARG	0.037
Eucl20D_ASP	0.203
Eucl20D_CYS	-0.228
Eucl20D_GLN	0.195
Eucl20D_GLY	0.158
Eucl20D_ILE	0.282
Eucl20D_LEU	-0.244
Eucl20D_LYS	0.254
Eucl20D_MET	0.217
Eucl20D_PHE	0.205
Eucl20D_PRO	0.215
Eucl20D_SER	0.070
Eucl20D_THR	0.331
Eucl20D_TRP	-0.045
Eucl20D_TYR	-0.102
Eucl20D_VAL	-0.092
EntropySubEucl	6.271
EntropySuperEucl	-20.377
RelEntropySubEucl	14.979
RelEntropySuperEucl	-25.650
HydroAvgEucl	0.130
HydroWToverAvgEucl	0.518
HydroMutoverAvgEucl	-0.729
PolarEucl	0.009
ChargedEucl	0.043
PosEucl	0.191
RelSolvAccessEucl	5.803
BfactorEucl	0.032
normBfactorEucl	0.002
sBfactorEucl	-0.012
snormBfactorEucl	-0.075
Eucl2FT	0.053
Eucl2Ligand	-0.070
Eucl2FTLigand	0.073
Hbond_6A	-0.028
SaltBridge_6A	0.226
SaltBridge_2layers	-0.569
