# Supplementary material for: Neighborhood Properties Are Important Determinants of Temperature Sensitive Mutations
Source: PLoS One. 2011 Dec 2;6(12):e28507. doi: 10.1371/journal.pone.0028507 (PMC3229608; doi:10.1371/journal.pone.0028507)
Supplement: Table S9 — The “topological neighborhood” model. (PDF) [file pone.0028507.s010.pdf]

Table S9 - The "topological neighborhood" model

Feature	Estimate
(Intercept)	1.808
DT20D_C	-0.550
DT20D_D	0.048
DT20D_F	0.223
DT20D_H	-0.153
DT20D_I	0.164
DT20D_K	0.590
DT20D_L	-0.199
DT20D_M	0.156
DT20D_N	-0.163
DT20D_P	0.192
DT20D_Q	0.348
DT20D_S	-0.010
DT20D_T	0.185
DT20D_V	-0.273
DT20D_W	0.110
DT20D_Y	-0.146
DTcontact	0.003
EntropySubDT	-8.289
EntropySuperDT	-2.316
RelEntropySuperDT	-6.448
HydroAvgDT	0.086
HydroWToverAvgDT	0.138
HydroMutoverAvgDT	-0.594
ChargedDT	0.173
SolvAccessDT	-0.043
RelSolvAccessDT	5.938
BfactorDT	0.005
DTcount	0.026
DTcountType0	0.027
DTcountType1	0.109
DTcountType2	0.034
DTcountType4	-0.087
