# Supplementary material for: Neighborhood Properties Are Important Determinants of Temperature Sensitive Mutations
Source: PLoS One. 2011 Dec 2;6(12):e28507. doi: 10.1371/journal.pone.0028507 (PMC3229608; doi:10.1371/journal.pone.0028507)
Supplement: Table S10 — The “all features” model. (PDF) [file pone.0028507.s011.pdf]

Table S10 - The “all features” model

Feature	Estimate
(Intercept)	-3.840
EntropySub	-0.813
EntropySuper	0.416
RelEntropySub	1.127
PHC	0.552
VolumeDiff	0.003
ChargeDiff	-0.145
Grantham	0.002
Unusual	0.417
NonPolarMut	0.197
NonPolar2Polar	-0.002
Polar2Charged	-1.039
Polar2Polar	0.004
Charged2Charged	-0.542
DisorderRegion	2.146
SolvAccessWT	0.008
SolvAccessDiff	-0.002
RelSolvAccessWT	-4.113
RelSolvAccessMut	-0.064
BuryWT	1.057
BuryMut	0.488
IsLigand	0.165
InStruct	0.448
HelixBreaker	-0.851
ddGPoPMuSiC	0.215
ddGratioFoldX	0.836
AA20D_A	0.019
AA20D_C	0.062
AA20D_D	-0.040
AA20D_F	0.059
AA20D_G	0.012
AA20D_H	-0.166
AA20D_L	-0.350
AA20D_M	-0.042
AA20D_P	-0.130
AA20D_Q	-0.069
AA20D_S	-0.135
AA20D_T	-0.249
AA20D_V	-0.170
RelEntropySubAA	0.806
PolarAA	-0.094
NonpolarAA	-0.134
ChargedAA	-0.068
NegAA	0.231
AA2FTLigand	0.013
HydroMomentMut	-0.002
HydroMomentDiff	-0.004
SolvAccessAA	-0.013
sBfactorAA	0.009
snormBfactorAA	0.002
Eucl20D_ALA	0.120
Eucl20D_ASN	-0.082
Eucl20D_ASP	0.004
Eucl20D_CYS	-0.134
Eucl20D_GLU	-0.070
Eucl20D_GLY	0.047
Eucl20D_ILE	0.193

Eucl20D_LEU	-0.150
Eucl20D_LYS	0.057
Eucl20D_MET	0.147
Eucl20D_PRO	0.097
Eucl20D_SER	-0.020
Eucl20D_THR	0.264
Eucl20D_TRP	-0.331
Eucl20D_TYR	-0.148
Eucl20D_VAL	-0.023
RelEntropySubEucl	7.362
RelEntropySuperEucl	-2.927
HydroMutoverAvgEucl	-0.887
PosEucl	0.039
RelSolvAccessEucl	1.363
BfactorEucl	0.007
Eucl2FT	0.028
Eucl2Ligand	-0.003
Eucl2FTLigand	0.005
Hbond_6A	-0.039
SaltBridge_6A	0.185
Hbond_2layers	-0.010
SaltBridge_2layers	-0.510
DT20D_A	0.010
DT20D_C	-0.461
DT20D_D	0.207
DT20D_E	-0.040
DT20D_I	0.215
DT20D_K	0.081
DT20D_L	0.019
DT20D_N	-0.261
DT20D_Q	-0.024
DT20D_S	0.031
DT20D_T	0.008
DT20D_V	-0.186
DT20D_W	-0.109
DT20D_Y	-0.079
RelEntropySuperDT	-2.974
HydroAvgDT	0.016
RelSolvAccessDT	0.439
DTcountType0	0.043
DTcountType2	0.062
DTcountType3	-0.066
