# Supplementary material for: Neighborhood Properties Are Important Determinants of Temperature Sensitive Mutations
Source: PLoS One. 2011 Dec 2;6(12):e28507. doi: 10.1371/journal.pone.0028507 (PMC3229608; doi:10.1371/journal.pone.0028507)
Supplement: Table S11 — The “sequence features” model. (PDF) [file pone.0028507.s012.pdf]

Table S11 - The "sequence features" model

Feature	Estimate
(Intercept)	-1.042
EntropySub	-1.315
RelEntropySub	0.902
PHC	0.661
HydrophobMut	-0.010
HydrophobDiff	-0.005
ChargeDiff	-0.153
NonPolarWT	0.174
NonPolarMut	0.176
NonPolar2Charged	0.284
Polar2Charged	-0.613
Polar2Polar	-0.101
Charged2Charged	-0.205
DisorderRegion	1.560
AA20D_A	0.059
AA20D_E	-0.123
AA20D_G	0.103
AA20D_H	-0.371
AA20D_K	0.049
AA20D_L	-0.434
AA20D_M	-0.035
AA20D_S	-0.109
AA20D_T	-0.027
AA20D_V	-0.297
AA20D_W	-0.529
AA20D_Y	-0.036
EntropySubAA	-5.404
EntropySuperAA	5.262
RelEntropySubAA	0.106
RelEntropySuperAA	-0.246
HydroAvgWT	0.012
HydroAvgDiff	0.000
PolarAA	-0.017
NonpolarAA	-0.030
NegAA	0.146
