# Supplementary material for: Neighborhood Properties Are Important Determinants of Temperature Sensitive Mutations
Source: PLoS One. 2011 Dec 2;6(12):e28507. doi: 10.1371/journal.pone.0028507 (PMC3229608; doi:10.1371/journal.pone.0028507)
Supplement: Table S12 — The “structure features” model. (PDF) [file pone.0028507.s013.pdf]

**Table S12 - The “structure features” model**

| Feature | Estimate |
|---|---|
| (Intercept) | -3.952 |
| SolvAccessWT | 0.008 |
| SolvAccessMut | 0.004 |
| RelSolvAccessWT | -4.968 |
| RelSolvAccessMut | -0.675 |
| BuryWT | 1.131 |
| BuryMut | 0.301 |
| IsLigand | 0.318 |
| InStruct | 1.492 |
| HelixBreaker | -1.198 |
| Bfactor | 0.090 |
| sBfactor | -0.058 |
| snormBfactor | -0.008 |
| ddGPoPMuSiC | 0.384 |
| ddGratioFoldX | 1.362 |
| AA2FT | 0.002 |
| AA2Ligand | -0.033 |
| AA2FTLigand | 0.050 |
| HydroMomentMut | -0.006 |
| HydroMomentDiff | -0.002 |
| SolvAccessAA | 0.012 |
| RelSolvAccessAA | -2.700 |
| sBfactorAA | 0.005 |
| snormBfactorAA | 0.019 |
| Eucl20D_ALA | 0.133 |
| Eucl20D_ASN | -0.067 |
| Eucl20D_ASP | 0.033 |
| Eucl20D_CYS | -0.058 |
| Eucl20D_GLN | 0.041 |
| Eucl20D_GLU | -0.070 |
| Eucl20D_GLY | 0.144 |
| Eucl20D_HIS | 0.082 |
| Eucl20D_ILE | 0.202 |
| Eucl20D_LEU | -0.249 |
| Eucl20D_LYS | 0.108 |
| Eucl20D_MET | 0.195 |
| Eucl20D_PHE | 0.099 |
| Eucl20D_PRO | 0.075 |
| Eucl20D_SER | -0.036 |
| Eucl20D_THR | 0.319 |
| Eucl20D_TRP | -0.188 |
| Eucl20D_TYR | -0.265 |
| Eucl20D_VAL | -0.071 |
| EntropySubEucl | 13.860 |
| EntropySuperEucl | -22.087 |
| RelEntropySubEucl | 22.839 |
| RelEntropySuperEucl | -25.571 |
| HydroAvgEucl | 0.030 |
| HydroWToverAvgEucl | 1.570 |
| HydroMutoverAvgEucl | -2.907 |
| PosEucl | 0.123 |
| SolvAccessEucl | 0.021 |
| RelSolvAccessEucl | -3.037 |
| BfactorEucl | 0.122 |
| normBfactorEucl | 0.001 |
| sBfactorEucl | -0.065 |
| snormBfactorEucl | -0.029 |

| | |
|---|---|
| Eucl2FT | 0.050 |
| Eucl2Ligand | -0.066 |
| Eucl2FTLigand | 0.071 |
| Hbond_6A | -0.006 |
| SaltBridge_6A | 0.180 |
| Hbond_2layers | -0.007 |
| SaltBridge_2layers | -0.643 |
| DT20D_A | -0.047 |
| DT20D_C | -0.859 |
| DT20D_D | 0.181 |
| DT20D_F | -0.001 |
| DT20D_G | 0.089 |
| DT20D_H | -0.276 |
| DT20D_I | 0.151 |
| DT20D_K | 0.463 |
| DT20D_L | -0.084 |
| DT20D_M | -0.056 |
| DT20D_N | -0.120 |
| DT20D_P | 0.104 |
| DT20D_Q | 0.148 |
| DT20D_S | 0.197 |
| DT20D_T | 0.013 |
| DT20D_V | -0.360 |
| DT20D_W | 0.030 |
| DT20D_Y | 0.095 |
| EntropySubDT | -0.174 |
| EntropySuperDT | -3.959 |
| RelEntropySubDT | 2.254 |
| RelEntropySuperDT | -6.224 |
| HydroAvgDT | 0.111 |
| HydroWToverAvgDT | -1.747 |
| HydroMutoverAvgDT | 2.459 |
| ChargedDT | 0.149 |
| NegDT | -0.082 |
| SolvAccessDT | -0.018 |
| RelSolvAccessDT | 8.095 |
| sBfactorDT | -0.063 |
| snormBfactorDT | -0.008 |
| DTcountType1 | 0.037 |
| DTcountType2 | 0.105 |
| DTcountType3 | -0.116 |
| DTcountType4 | -0.118 |
