# Supplementary material for: Neighborhood Properties Are Important Determinants of Temperature Sensitive Mutations
Source: PLoS One. 2011 Dec 2;6(12):e28507. doi: 10.1371/journal.pone.0028507 (PMC3229608; doi:10.1371/journal.pone.0028507)
Supplement: Table S13 — Performance of TS prediction models from a ten-fold cross-validation. (PDF) [file pone.0028507.s014.pdf]

Table S13 - Performance of TS prediction models from a ten-fold cross-validation

Model	Model evaluation*				
	ACC	MCC	AUC	KL	DD
Site features	0.77	0.38	0.85	0.17	0.45
Neighborhood features	0.83	0.47	0.88	0.08	0.68
Sequence neighborhood	0.79	0.37	0.82	0.11	0.49
Euclidean neighborhood	0.81	0.42	0.87	0.10	0.58
Topological neighborhood	0.79	0.40	0.85	0.13	0.53
All features	0.84	0.51	0.91	0.08	0.78
Sequence features	0.80	0.43	0.87	0.11	0.63
Structure features	0.83	0.48	0.90	0.08	0.73

* ACC = accuracy, MCC = Matthews correlation coefficient, AUC = area under the curve, KL = Kullback-Leibler divergence, DD = distribution distance.
